# Exposure assessment of process-related contaminants in food by biomarker monitoring

**DOI:** 10.1007/s00204-017-2143-2

**Published:** 2018-01-04

**Authors:** Ivonne M. C. M. Rietjens, P. Dussort, Helmut Günther, Paul Hanlon, Hiroshi Honda, Angela Mally, Sue O’Hagan, Gabriele Scholz, Albrecht Seidel, James Swenberg, Justin Teeguarden, Gerhard Eisenbrand

**Affiliations:** 1 Division of Toxicology, Wageningen University, Stippeneng 4, 6708 WE Wageningen, The Netherlands; 2 International Life Sciences Institute, Europe (ILSI Europe), Av E. Mounier 83, Box 6, 1200 Brussels, Belgium; 3 Mondelēz International, Postfach 10 78 40, 28078 Bremen, Germany; 4 Abbott Nutrition, 3300 Stelzer Road, Dept. 104070, Bldg. RP3-2, Columbus, OH 43219 USA; 5 KAO Corporation, R&D Safety Science Research, 2606 Akabane, Ichikai-Machi, Haga-Gun, Tochigi, 321 3497 Japan; 6 Department of Toxicology, University of Würzburg, Versbacher Strasse 9, 97078 Würzburg, Germany; 7 PepsiCo Europe, 4 Leycroft Road, Leicester, LE4 1ET UK; 8 Nestlé Research Center, Vers-chez-les-Blanc, PO Box 44, 1000 Lausanne 26, Switzerland; 9 Biochemical Institute for Environmental Carcinogens Prof. Dr. Gernot Grimmer-Foundation, Lurup 4, 22927 Grosshansdorf, Germany; 10 Environmental Science and Engineering, UNC-Chapel Hill Cancer Genetics, 253c Rosenau Hall, Chapel Hill, NC USA; 11 Pacific Northwest National Laboratory, P.O. Box 999, Richland, WA 99352 USA; 12 Division of Food Chemistry and Toxicology, Department of Chemistry, University of Kaiserslautern, P.O. Box 3049, 67653 Kaiserslautern, Germany

**Keywords:** Dietary process-related contaminants, Biomarkers, External exposure assessment, Physiologically based kinetic models, Risk assessment

## Abstract

Exposure assessment is a fundamental part of the risk assessment paradigm, but can often present a number of challenges and uncertainties. This is especially the case for process contaminants formed during the processing, e.g. heating of food, since they are in part highly reactive and/or volatile, thus making exposure assessment by analysing contents in food unreliable. New approaches are therefore required to accurately assess consumer exposure and thus better inform the risk assessment. Such novel approaches may include the use of biomarkers, physiologically based kinetic (PBK) modelling-facilitated reverse dosimetry, and/or duplicate diet studies. This review focuses on the state of the art with respect to the use of biomarkers of exposure for the process contaminants acrylamide, 3-MCPD esters, glycidyl esters, furan and acrolein. From the overview presented, it becomes clear that the field of assessing human exposure to process-related contaminants in food by biomarker monitoring is promising and strongly developing. The current state of the art as well as the existing data gaps and challenges for the future were defined. They include (1) using PBK modelling and duplicate diet studies to establish, preferably in humans, correlations between external exposure and biomarkers; (2) elucidation of the possible endogenous formation of the process-related contaminants and the resulting biomarker levels; (3) the influence of inter-individual variations and how to include that in the biomarker-based exposure predictions; (4) the correction for confounding factors; (5) the value of the different biomarkers in relation to exposure scenario’s and risk assessment, and (6) the possibilities of novel methodologies. In spite of these challenges it can be concluded that biomarker-based exposure assessment provides a unique opportunity to more accurately assess consumer exposure to process-related contaminants in food and thus to better inform risk assessment.

## Introduction

Exposure assessment is a fundamental part of the risk assessment paradigm but can often present a number of challenges and uncertainties. The science of exposure assessment is undergoing fundamental change with the development of new approaches that do not just focus on occurrence in food and consumption patterns, i.e. on external exposure metrics. The advent of metabolomics, picturing the totality of metabolite profiles opens up the prospect of collecting comprehensive exposure information on specific dietary chemicals and their interaction in the body. Another option consists of monitoring selected biomarkers in body fluids or tissues as quantitative exposure indicators, based on detailed knowledge of the metabolism of the dietary chemical in focus. This allows determination of external exposure levels based on these biomarkers of internal exposure, as opposed to less reliable methods such as food frequency questionnaires.

Deriving accurate exposure estimates for certain contaminants in food can be particularly challenging. This is especially the case for several process-related contaminants. Process contaminants are formed during the processing, e.g. heating of food. They can be formed during both home cooking and industrial food manufacturing. Some are known to be volatile, e.g. furan, or to bind to food constituents. An example of this latter aspect is acrylamide that has been shown to bind, depending on storage time and conditions, to some degree covalently to insoluble matrix constituents of roast and ground coffee thus being no longer bioavailable (Baum et al. 2008). Assessing how much of a process contaminant such as acrylamide is generated through home cooking can also be challenging because these conditions are very variable. In the case of furan, preparation steps, from cooking food at home and preparation of beverages like coffee to processing food samples in the analytical laboratories can have an impact on the levels of furan detected in the food or beverage. There is also evidence to suggest that acrylamide and other process-related contaminants are formed endogenously. Therefore, there are significant uncertainties related to the assessment of consumer exposure to process-related contaminants.

Due to these uncertainties, assessing exposure from dietary and environmental sources only may underestimate the true exposure. New approaches are therefore required to accurately assess consumer exposure and thus better inform the risk assessment of the exposure to these contaminants. Such novel approaches may include use of biomarkers, physiologically based kinetic (PBK) modelling-facilitated reverse dosimetry, and/or duplicate diet studies and the use of stable isotope labelled marker compounds.

This review focuses on the state of the art with respect to the use of biomarkers of exposure for the process contaminants acrylamide, 3-MCPD esters, glycidyl esters, furan and acrolein. These are all process contaminants of public health concern that have undergone or are undergoing safety assessments by risk assessment bodies such as the European Food Safety Authority (EFSA) or the Joint FAO/WHO Expert Committee on Food Additives (JECFA). These compounds have the potential to impact public health based on risk assessments indicating low safety margins between human exposure and adverse effect levels observed in animal studies. Risk assessments prepared on acrylamide by the EFSA and the JECFA indicate the need for more accurate exposure estimates utilizing biomarkers. In its assessment of acrylamide in food JECFA recommended that, in longitudinal studies of acrylamide and glycidamide, haemoglobin adducts are measured over time in relation to concurrent dietary exposures (JECFA 2011). EFSA recommended that in order to improve the exposure assessment of acrylamide duplicate diet studies be conducted and that data on urinary metabolite levels be collected from individuals participating in such studies for the purpose of validating the biomarkers (EFSA 2015). Because of its volatility, establishing accurate estimates of exposure to dietary furan will be difficult and associated with a large amount of uncertainty. Acrolein is formed during cooking, particularly when oil is overheated. It can be found in fried food, cooking oil and roasted coffee (lARC 1993). Contents in food are often determined by headspace techniques which, however, only measure the volatile proportion of acrolein. Because of its high Michael reactivity, acrolein is supposed to rapidly react with nucleophilic food constituents, including water, forming non-volatile acrolein adducts (Abraham et al. 2011) Moreover, acrylamide and acrolein are also formed endogenously within the body (Uchida et al. 1998; Stevens and Maier 2008; Goempel et al. 2017) and therefore make interesting case studies on how to differentiate between exposure from exogenous and endogenous sources. Duplicate diet studies are promising since they allow to exactly measure the dietary intake of a given process related contaminant and to compare this with the urinary output of appropriate metabolites that allow biomarker-based dosimetry. This, however, may not be helpful in the case of acrolein unless methods become available to comprehensively analyse free acrolein together with that reversibly bound to food matrix constituents.

The review shortly introduces how and where these process contaminants are formed and their potential impact on human health. Then the current knowledge on urinary, blood and tissue biomarkers for each of these contaminants is summarized and where available, PBK models are discussed. The potential for endogenous formation of these process contaminants is also considered. There are clearly data gaps for most of these process contaminants that would need to be addressed in order to provide reliable and reproducible quantitative estimates of exposure via the use of their biomarkers. Ultimately the aim would be to incorporate such quantitative exposure estimates into risk assessment to better define the public health risk and inform risk management action.

## State of the art

### Acrylamide

#### Characterization, formation, occurrence and public health concern

Acrylamide (AA) is a large volume industrial chemical used as monomeric building block in the production of polyacrylamides. For analytical purposes it serves worldwide for in situ preparation of polyacrylamide gels in biochemical laboratories. In 2002, AA surprisingly was reported by Swedish scientists to be present in many commonly consumed foods as a result of heat treatment (Rosén and Hellenäs 2002). AA is a process related food contaminant generated during various food heating processes from reducing carbohydrates and asparagine. Due to the natural presence of these precursor constituents in foods, the most important mechanism of AA formation in foods appears to result from the heat induced reaction of such precursors in the course of Maillard reactions governing food browning, involving the intermediate formation of 3-aminopropionamide and its equivalents. As a consequence, substantial AA contamination has consistently been found in strongly heated carbohydrate-rich foods such as French fries and potato chips or crisps, and roasted coffee beans (Guth et al. 2013; EFSA 2015). In lipid-rich food the route of AA formation via acrolein and ammonia may also play some role, as acrolein is formed in substantial amount from glycerol and mono-acylglycerols upon heating of animal and vegetable fats (Umano and Shibamoto 1987; Gertz and Klostermann 2002; Yasuhara et al. 2003).

AA has been classified by the International Agency for Research on Cancer (IARC) (IARC 1994) as genotoxic and probably carcinogenic to humans (group 2A). Dietary exposure of adults in Europe has been estimated at the 95th percentile between 0.6 and 2.0 µg/kg body weight (bw)/day and for children between 1.4 and 3.4 µg/kg bw/day (EFSA 2015).

EFSA calculated margins of exposure (MOE) ranging from 283 to 50 for the 95th percentile average exposure estimates, based on a benchmark dose lower confidence limit (BMDL10) of 0.17 mg/kg bw/day. This BMDL10 was deduced from a 2-year National Toxicology Program (NTP) dose–response study in male B6C3F1 mice, taking Harderian gland neoplasms as the critical neoplastic lesion. Based on this rather low MOE range resulting from the above exposure estimates, EFSA expressed a human health concern with respect to neoplastic effects (EFSA 2015). No concern was expressed with respect to neurotoxicity or other adverse effects potentially associated with dietary exposure to AA. However, the relevance of genotoxic efficacy of AA to cancer endpoints observed in animals is under continuous scrutiny. For instance the plausibility of hypothesized modes of action (MOAs) of AA causing thyroid (but not liver) tumours in rats, namely genotoxicity/mutagenicity versus thyroid hormone dysregulation, was tested in F344 rats at dosages of up to 12.0 mg/kg bw/day for different subchronic time periods. Differently expressed genes in both tissues provided at best marginal support for hormonal and genotoxic MOAs. Instead, pronounced effects on calcium signalling/cytoskeletal genes were seen in the thyroid target organ, suggesting perturbation of calcium signalling as a novel MOA for AA mediated thyroid carcinogenicity in the male rat (Chepelev et al. 2017). A similar MOA has also been evidenced as probably causative for tumour induction in another target organ of AA, the rat testes, under the same experimental conditions (Recio et al. 2017). Differential mutagenicity studies in the micronucleus (MN) and *Pig*-a gene mutation assays in F344 rats and B6C3F1 mice, performed under similar experimental conditions, revealed negative or equivocal results, except for MN induction in mice, supporting structural DNA damage but not point mutations as MOA underlying genotoxicity. Moreover, a lack of in vivo genotoxicity observed at dosages < 6.0 mg/kg/day was found consistent with the notion that non-genotoxic mechanisms contribute to AA induced carcinogenicity in rodents (Hobbs et al. 2016).

#### Urinary biomarkers

**Fig. 1:**
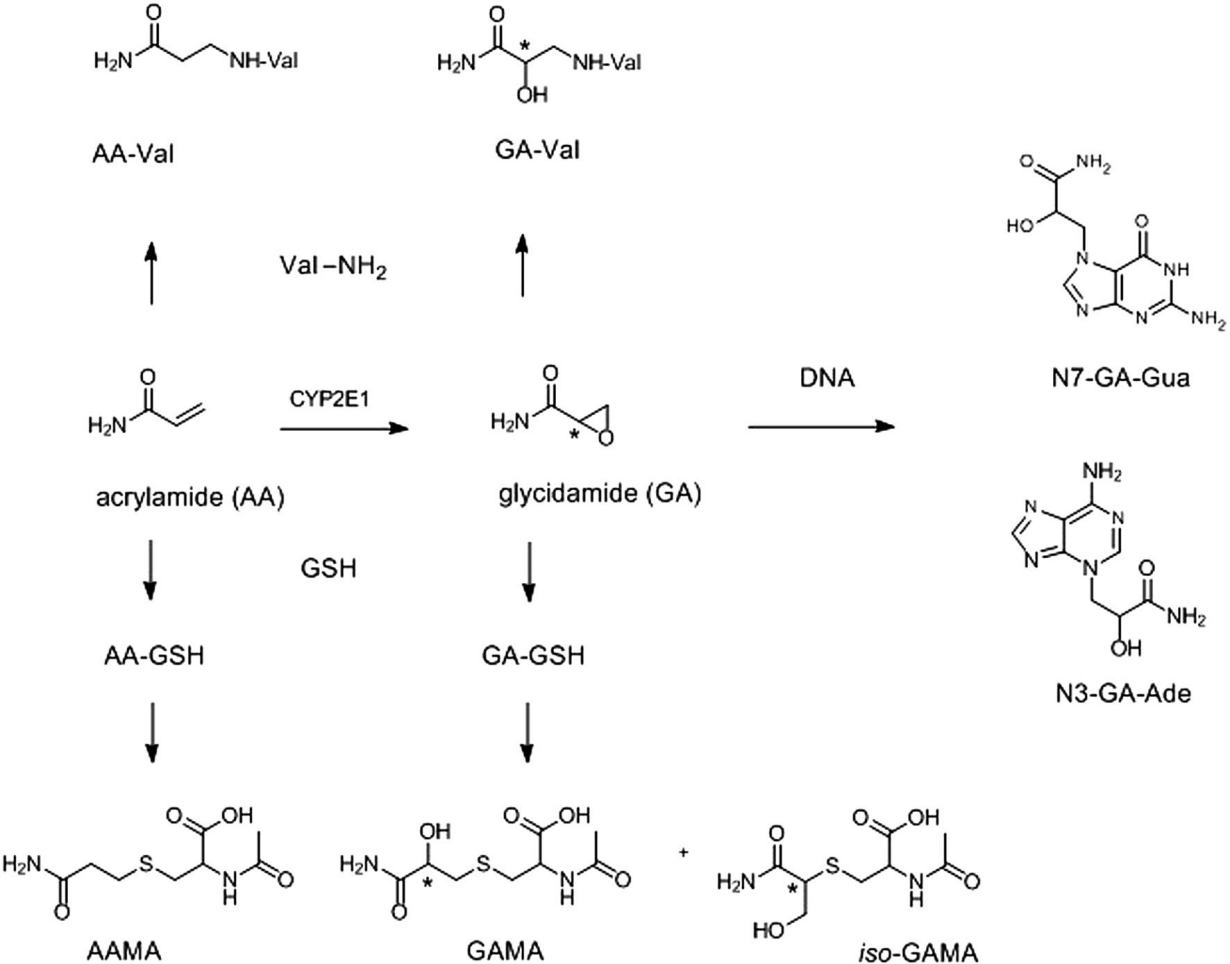
Biotransformation of AA and biomarkers of AA exposure including the AA- and GA-related urinary mercapturic acids, *N*-acetyl-*S*-(2-carbamoylethyl)-l-cysteine (AAMA) and *N*-(R,S)-acetyl-*S*-(2-carbamoyl-2-hydroxyethyl)-l-cysteine (GAMA), *N*-(R,S)-acetyl-*S*-(1-carbamoyl-2-hydroxyethyl)-l-cysteine (*iso*-GAMA), and *N*-(2-carbamoylethyl)valine (AA-Val) and *N*-(2-carbamoyl-2-hydroxyethyl)valine (GA-Val) reflecting Hb adduct formation, as well as the formation of depurinating DNA adducts by GA at N7 of guanine (N7-GA-Gua)(major) and N3 of adenosine (N3-GA-Ade)(minor). The human metabolite AAMA Sulfoxide (AAMA-sul) is not indicated

AA is rapidly absorbed from the gastrointestinal tract and undergoes fast systemic distribution and extensive biotransformation. It reacts with plasma and other proteins, preferably at nucleophilic thiol or amino groups and also is efficiently coupled to glutathione (GSH) during first pass metabolism in the liver. Importantly, it undergoes cytochrome P450 2E1-dependent conversion into the ultimate genotoxic metabolite 2,3-epoxypropanamide or glycidamide (GA) (Fig. 1). Interspecies differences, dose dependencies and impact of linear versus nonlinear effects result in differences in epoxidation of AA to GA (Gargas et al. 2009). Like AA, GA avidly forms covalent adducts with proteins and GSH but may also react with DNA, generating DNA adducts, preferentially at N7 of deoxyguanine forming a depurinating guanosine adduct (N7-GA-Gua) (Fig. 1). Thus, GA formation and its reaction with DNA are conceived as the key events underlying genotoxicity and carcinogenicity of AA (EFSA 2015). The primary glutathione conjugates of AA and GA (AA-GSH and GA-GSH) are further metabolically processed, mainly in the kidney and urinary tract and excreted in urine as AA- and GA-related mercapturic acids (MA), *N*-acetyl-*S*-(2-carbamoylethyl)-l-cysteine (AAMA), *N*-(R,S)-acetyl-*S*-(2-carbamoyl-2-hydroxyethyl)-l-cysteine (GAMA), and *N*-(R,S)-acetyl-*S*-(1-carbamoyl-2-hydroxyethyl)-l-cysteine (*iso*-GAMA) (Hartmann et al. 2009). Humans were found to also excrete a sulfoxide of AAMA (AAMA-sul), to an extent comparable to GAMA (Wang et al. 2017).

In primary rat hepatocytes the rate of AA-GSH adduct formation was found to be up to 3 times higher than the formation of the genotoxic epoxide GA under low exposure conditions (2 µM AA in the incubation medium, mimicking worst case consumer exposure) (Watzek et al. 2012a). Urinary AAMA and GAMA have proven useful as valid biomarkers for AA exposure (Bjellaas et al. 2005, 2007; Boettcher et al. 2005; Kellert et al. 2006; Urban et al. 2006). Studies on toxicokinetics of AA in humans report roughly half of an ingested AA dose to be eliminated as AAMA, whereas 5–6% is excreted in the urine as GAMA (Boettcher et al. 2006; Fennell et al. 2006; Fuhr et al. 2006; Hartmann et al. 2008). The kinetics of mercapturic acid excretion in urine after AA exposure shows AAMA/GAMA to be rapidly formed in humans and excreted nearly quantitatively within 72 h (Ruenz et al. 2016). Monitoring of urinary AAMA/GAMA therefore is ideally suited to assess controlled short-term exposure as executed in dietary intervention studies. For such studies, duplicate diet methodology has been recommended as the method of choice to achieve exactly controlled dietary AA intake under controlled conditions (EFSA 2015; Ruenz et al. 2016). This methodology enables reliable dosimetry of the AA ingested by the study volunteers because AA contents are measured in duplicates of the prepared meals on the plate. Thus correlations between controlled dietary AA exposure and biomarker kinetics can be investigated under realistic conditions (Ruenz et al. 2016; Goempel et al. 2017). Monitoring the urinary AAMA/GAMA excretion rates can inform about the balance within the individual metabolic setup governing AA detoxification by direct or glutathione *S*-transferase-mediated GSH coupling and AA toxification by phase I epoxidation to GA, because the latter is, similar to AA, also subject to subsequent phase II biotransformation into GAMA.

#### Blood biomarkers

During their systemic distribution and biotransformation AA and GA react with nucleophilic sites throughout the body. In blood, a major reaction consists in formation of covalent adducts with amino and thiol groups of haemoglobin (Hb). The Hb-adducts, *N*-(2-carbamoylethyl)valine (AA-Val) and *N*-(2-carbamoyl-2-hydroxyethyl)valine (GA-Val) (Fig. 1) can specifically be determined with a modified Edman degradation of the alkylated N-terminal valine and a subsequent acetonization of the glycidamide-valine pentafluorophenylthiohydantoin derivative for determination by GC/MS analysis (Paulsson et al. 2003a). More recently, an alternative method using liquid chromatography–tandem mass spectrometry (LC–MS/MS) for simultaneous determination of AA-Val and GA-Val has been reported (Von Stedingk et al. 2010). This rapid and very sensitive variant of the Edman procedure uses fluorescein isothiocyanate (FITC) as a novel Edman reagent. Prior to LC-MS/MS analysis the two fluorescein thiohydantoin derivatives of AA-Val and GA-Val are enriched and purified on SPE mixed mode anion exchange cartridges (Von Stedingk et al. 2010). Hb-adduct monitoring is considered a valuable method to assess the internal burden associated with longer term AA exposure, reflecting the exposure over the last 4 months, since the average lifespan of human erythrocytes is 120 days (Schettgen et al. 2004; Vesper et al. 2006; Goempel et al. 2017).

In laboratory animals GA-haemoglobin adduct concentrations have been reported to correlate with GA-DNA adduct levels found in the liver and therefore Hb-adducts have been proposed as surrogate biomarkers for DNA adduct formation (Tareke et al. 2006; Angerer et al. 2008; Hartmann et al. 2008). However, the correlation between GA-Hb-adducts and DNA adduct formation observed in animal experiments may not hold for low exposure situations close to human dietary intake levels. For instance, in Sprague Dawley rats ingesting AA (100 µg/kg bw/day) for 9 days in their feed, the biomarker response clearly indicated metabolic formation of GA as reflected by excretion of AAMA and GAMA (urinary GAMA/AAMA ratio about 0.5–1.1). However, Hb-adducts behaved quite differently: whereas there was a distinct cumulative formation of AA-Val over the whole exposure period, the biomarker indicative for GA in the blood did not deviate significantly from background. This suggested that any GA formed during first pass in the liver of these rats was efficiently GSH-coupled and detoxified. Indeed, GA was barely detectable in the blood (*C*_max_ < 0.06 µM), whereas AA reached substantial blood levels (0.5–2 µM) after intake from feed or water (Berger et al. 2011). The behaviour observed for GA-Hb-adducts would suggest that significantly enhanced genotoxicity may not be expected during first pass at this relatively low exposure level. This corollary was substantiated later by results of a dose–response study in rats with single oral AA doses of 0.1–10 000 µg/kg bw with monitoring excretion of urinary AAMA/GAMA and formation of N7-GA-Gua adducts in different organs. In the low dose range (0.1/1.0/10/–100 µg/kg bw) DNA adduct levels did not show linear dose dependence and remained in a very low background range, not exceeding 2 adducts/10^8^ nucleotides (Watzek et al. 2012b).

In a human biomarker monitoring study, 45 males and 46 females of the general population (aged from 6 to 80, median 36 years) were analysed simultaneously for Hb-adducts and urinary AAMA/GAMA. For Hb-adducts, the ratio between GA-Val and AA-Val was found individually highly variable (range 0.4–2.7, median 1.1) and roughly in a similar range as reported in a series of other international studies (0.5–1.1, median levels) (Hartmann et al. 2008). In laboratory animals, ratios of 1.8 for rats and of 5.4 for mice were reported, respectively, as compared to 1.0 for humans (Paulsson et al. 2003b). This indicates considerably higher rates of activating biotransformation of AA to GA in mice as compared to rats or humans. This conclusion was substantiated in later studies at a higher dose range (Tareke et al. 2006). The ratio of AA-Val to GA-Val after oral application of 50 mg/kg bw to rats was reported to be 0.38 (Sumner et al. 2003). By comparison, the ratio found in humans after intake of 0.5, 1.0, and 3 mg/kg AA was between 0.36 and 0.44 (Fennell et al. 2004).

#### Tissue biomarkers

Theoretically, in tissues covalent coupling of AA and GA to structural proteins may be exploitable for assessment of tissue exposure. However, adequate methodology has not been developed yet. An alternative approach lies in the determination of DNA lesions resulting from covalent binding of GA to DNA bases. In adult mice, the formation of glycidamide-guanine DNA (N7-GA-dG) adducts was found to represent by far the major type of DNA lesion, whereas glycidamide-adenosine DNA (N3-GA-Ade) adducts were detected as minor DNA lesion (2 orders of magnitude lower). Data were reported for liver, lung, and kidney after treatment with 50 mg AA/kg bw (Gamboa da Costa et al. 2003). The formation of N7-GA-dG adducts (1 adduct/10^8^ nucleotides) in liver of Fischer 344 rats given single oral doses of 100 μg/kg bw in drinking water (gavage) or via diet has also previously been reported (Doerge et al. 2005). In an extended dose–response study in female Sprague Dawley rats in vivo, formation of N7-GA-dG was measured 16 h after oral dosage of AA (0.1–10 0000 μg/kg bw) which was previously ascertained to represent *T*_max_ of N7-GA-dG levels in all tissues analysed (Watzek et al. 2012b). Background exposure to AA was minimized by applying, for 20 days before start and throughout the study, an experimental diet with an AA content of < 0.5 µg/kg. Urinary GAMA and AAMA were monitored at the same time point where N7-GA-Gua dosimetry was carried out, although at 16 h, formation and excretion of GAMA and AAMA were not yet complete, reaching a mean of 37.0 ± 11.5% of a given AA dose, with a GAMA/AAMA ratio of 0.4 ± 0.1. This ratio remained constant over the whole dose range with no indication of a dose-related shift.

At a dose of 1 μg AA/kg bw, the formation of N7-GA-dG adducts became detectable in kidney and lung (each with *p* < 0.001) but not in liver. At a 10-fold higher dose (10 μg/kg bw), adduct levels remained at the same low level, with 0.4, 1.1, and 0.7 N7-GA-dG/10^8^ nucleotides, respectively, in liver, kidney, and lung. At the next higher dose (100 μg/kg bw) increased adduct levels (*p* < 0.001) in all organs were measured but this increase was not dose related in a linear fashion, remaining at a very low level (≤ 2 N7-GA-dG/10^8^ nucleotides). Altogether, within the above low exposure range adduct formation up to 100 µg AA /kg bw was not linearly dose related and did not exceed a level of about 2 N7-GA-dG/10^8^ nucleotides. Conversely, in the higher dose range adduct levels were clearly dose dependent (Watzek et al. 2012b).

These experimental results may be put into perspective by comparison with background levels of DNA lesions reported for human tissues. For instance, 7-(2′-carboxyethyl)-dG, an adduct chemically similar to N7-GA-dG may arise from exposure to acrolein/acrylic acid. A level of 7.5/10^8^ nucleotides was measured in human liver (Cheng et al. 2010). Another comparison relates to human background levels of another structurally related N7 adduct, 7-(2-hydroxyethyl)-dG, considered to result predominantly from endogenous generation of ethane and ethylene oxide and the interaction of the latter with DNA. For human lymphocytes and liver tissue, levels of 48–58 adducts/10^8^ nucleotides have been reported (Wu et al. 1999). It needs to be ascertained whether indeed the above group of N7 adducts (and potentially some others) is comparable in terms of biological activities, thus potentially providing a perspective of grouping them together in a read across approach. It may be concluded here that single oral AA application in the dose range up to 100 µg/kg bw to rats resulted in tissue adduct levels considerably lower than reported for human background levels of structurally related N7-dG adducts.

#### Available PBK studies

In a model developed for AA, GA and their glutathione conjugates, including Hb-adducts and DNA adducts (liver), serum AA and GA levels were combined with urinary elimination levels for all components from rats and mice and simulated from intravenous and oral administration of 100 µg/kg AA or 120 µg/kg GA. Adduct formation and degradation rates were determined from 6 weeks exposure to approximatively 1 mg/kg AA in drinking water, followed by subsequent 6 weeks non exposure. In brief, simulations were performed based on exposure and elimination data available at that time from the literature (Fennell et al. 2004; Fuhr et al. 2006) in combination with data on AA and GA-Hb-adduct levels associated with “background” dietary exposure to AA in the general human population (Boettcher et al. 2005) to estimate human dietary exposure doses and associated human liver GA-DNA adduct levels. Human dietary “background” exposure was estimated by this approach to be about 0.2 µg/kg AA. This exposure was predicted to be associated with a steady-state human liver DNA adduct level of about 0.06–0.26/10^8^ nucleotides of GA-DNA adducts (Young et al. 2007).

In another study, the relationship between the toxifying and detoxifying (termed “oxidative” and “reductive”, respectively) metabolic pathways of acrylamide (AA) in the non-smoking general population was investigated by simultaneously monitoring Hb-adducts in blood and GAMA/AAMA in the urine (Hartmann et al. 2008). The biomarker levels were used to calculate the daily AA intake. In brief, on the basis of the AA-Val concentrations in relation to the adduct formation reaction rate constant, (*k* = 4.4 × 10^−6^ L g of globin^−1^ h^−1^) and the mean erythrocyte lifespan (120 days), the value for the area under the curve (AUC) of each person was calculated. This was multiplied by the elimination rate constant (*E*_*k*_) in humans of 0.15 h^−1^ (Calleman 1996) and the volume of distribution (VD) of 0.38 L/kg (Fennell et al. 2004). This allowed to calculate an estimate of the daily intake of AA, in a similar way as already approached in earlier studies (Calleman 1996; Schettgen et al. 2003).

In a comprehensive study in non-smoking postmenopausal female controls from nested ovarian and endometrial case-control studies investigated within the EPIC cohort (416 and 386 controls, respectively), the median (25th–75th percentile) estimated dietary AA intake was 0.3 (0.2–0.5) μg/kg bw/day and the median AA-Hb and GA-Hb adduct levels were 41.3 (32.8–53.1) and 34.2 (25.4–46.9) pmol/g of Hb, respectively, at baseline. A moderate proportion of acrylamide adduct variation, in terms of the ratio of GA-Hb/AA-Hb could be ascribed to alcohol intake and body mass index [never versus ever drinkers, *p* value < 0.0001; < 25, 25 to < 30 (Obón-Santacana et al. 2017)].

On the basis of the above model a median daily intake of 0.43 μg/kg bw/day (range 0.21–1.04 μg/kg bw/day) was calculated. Of note, according to this model, children were calculated to ingest 1.3–1.5 times more AA per kilogram of body weight than adults. Also, the GAMA/AAMA ratio was found to be significantly higher in the group of young children (6–10 years) with a median level of 0.5. Gender-related differences in internal exposure and metabolism were not observed. The latter finding was subsequently confirmed by a study that likewise did not find internal exposure to acrylamide, as monitored by Hb-adducts, to be affected by gender (Vesper et al. 2010). In contrast, in Chinese adolescents (about 100 college students) faster urinary excretion kinetics of AAMA (*T*_1/2_ = 12.3 h and *C*_max_ = 1973.4 nmol/g urinary creatinine) in women as compared to men (*T*_1/2_ = 20.0 h and *C*_max_ = 1044.7 nmol/g urinary creatinine) was observed. In addition, higher *C*_max_ and AUC values of all GAMA and AAMA metabolites in urine of normal weight subjects (BMI < 24) have been found, as compared to those with higher BMI (Wang et al. 2017).

#### Endogenous formation and accompanying levels of the biomarkers

Some observations from human studies appeared to indicate higher daily intake estimates of acrylamide based on Hb-adduct levels as compared to estimates from dietary questionnaires (Tareke et al. 2008). It was thus hypothesized that the Maillard reaction between reducing carbohydrates and asparagine also takes place endogenously under physiological conditions. To some extent, this may contribute to endogenous acrylamide generation (Mottram et al. 2002; Stadler et al. 2002; Baynes et al. 2005). Another potential pathway of endogenous AA formation was suggested by the finding of increased acrylamide and GA-Hb adduct levels in mice treated with chemicals that induced oxidative stress. Based on this observation it was hypothesized that acrylamide may also be formed to some extent as a result of oxidation (Tareke et al. 2008).

The assumption of an endogenous AA background was substantiated by observations in animal experiments and in human intervention studies under tightly controlled conditions. For instance, in an extended dose–response study, rats were held under environmental and dietary conditions of minimized AA exposure for 20 days before the onset of the experiment and throughout the whole study. In the untreated controls a background urinary excretion of about 0.8 nmol (cumulative excretion 16 h post-application) of GAMA and AAMA was consistently found, equivalent to an internal exposure estimate of about 1.6-2 nmol (0.6–0.7 μg/kg bw) of ingested AA. This was considerably more than was to be expected from an assumed worst case AA intake via low levels in the feed of 0.4 nmol AA per day (0.1 μg/kg bw) (Watzek et al. 2012a). Furthermore, a human intervention study was performed with 14 healthy male volunteers over a period of 9 days, under controlled conditions, minimizing any inadvertent AA exposure. Dietary exposure to AA was measured by determining AA contents in duplicates of all meals consumed. The study design included an initial washout period of 3 days on AA-minimized diet, resulting in dietary AA exposure not exceeding 41 ng/kg bw/day. At the end of the initial 3-day washout period, an AAMA baseline level of 93 ± 31 nmol/day was recorded. Assuming 30% of AA excreted within 24 h as AAMA, this would suggest a baseline endogenous exposure level to AA (on washout day 2) equivalent to 0.2–0.3 μg AA/kg bw/day exogenous exposure (Ruenz et al. 2016). A subsequent duplicate diet study was undertaken to confirm this finding and to also address the question whether it can be attributed indeed to endogenous background AA exposure in humans or whether delayed AAMA release from some deep compartment may play a role (e.g. by potential cleavage from some structural or plasma protein). To this end an extended duplicate diet study, encompassing washout periods of up to 13 days was carried out in 12 human volunteers. Half of the volunteers (*n* = 6) ingested ^13^C_3_D_3_-AA (1 µg/kg bw), the other half consumed freshly prepared meals with exactly known AA content by duplicate analysis. At the end of the 13-day washout period the ^13^C_3_D_3_-AA group excreted an unlabelled AAMA baseline level of 0.14 ± 0.10 µmol/day, although AA intake was only about 0.06 µmol/day. This sustained over-proportional AAMA background indicated an endogenous AA baseline exposure level of 0.3–0.4 µg/kg bw/day. The excretion of ^13^C_3_D_3_-AA was practically complete within 72–96 h, thus ruling out delayed release of AA (or any other GAMA/AAMA precursor) from deep body compartments. These results provide compelling support for the hypothesis of a sustained endogenous AA formation in the human body (Goempel et al. 2017).

#### Data gaps and research needs

The database on dietary AA exposure and its association with urinary and/or blood exposure biomarkers needs to be substantiated to the extent to provide a solid basis for reverse dosimetry of dietary AA exposure. This should also encompass an in depth characterization of inter-individual variance in expression and activity of relevant biotransformation enzymes, under due consideration of polymorphisms as well as age and gender-related variances. Such studies will require tightly controlled conditions of dietary AA exposure, as exemplified by duplicate diet studies. Short-term biomarkers like urinary GAMA and AAMA and their ratio (GAMA/AAMA) appear to be most appropriate. Hb-adducts are known to reflect factors of influence associated with long term exposure. Since Hb-adducts have been proposed as surrogate markers for genotoxicity of GA, it will be of great importance to further investigate whether this surrogate biomarker function indeed holds true for the dietary low exposure range. This needs to be achieved by monitoring steady-state levels of Hb-adducts and N7-GA-dG DNA adducts simultaneously in blood under tightly controlled longer term exposure conditions. However, besides the logistic difficulties of maintaining tightly controlled exposure conditions for the required extended time periods, the vastly different kinetics of the respective blood biomarkers need to be taken into consideration in order to establish correlations. Therefore simultaneous monitoring of urinary GAMA and AAMA as second short term biomarker may be a prerequisite. Such studies also need control groups of lowest achievable exposure (verified by duplicate diet) which adds to their complexity. The data will be very valuable to substantiate the reported findings on endogenous background levels of AA exposure which also will most probably be subject to individual or health/lifestyle associated variability.

### 3-MCPD

#### Characterization, formation, occurrence and public health concern

Monochloropropane-1,2-diols (MCPDs) are contaminants that are created during food production, especially in cases where foods containing fat and salt are processed at high temperatures (Crews et al. 2013). They can be chlorinated in the 2- or 3-position (2- and 3-MCPD, respectively). 3-MCPD was originally identified at high levels in acid hydrolysed vegetable proteins and soy sauce, but can be found in many food types (European Commission 2004). Analytical data on 2-MCPD occurrence are scarcer. 2-MCPD may occur in processed vegetable oils and foods that use processed vegetable oils as ingredients (Tennant and Gosling 2015), 3-MCPD is primarily found as fatty acid esters in a mixture of both, mono- and di-esters (MacMahon et al. 2013). The current understanding, based on the results from a limited number of 3-MCPD esters, is that enzymatic hydrolysis of the esters occurs at a significant rate in vivo, resulting in close correlations between the potency of free versus esterified 3-MCPD (Abraham et al. 2013). In the current risk assessment it is considered that both bound (in fatty acid esters) and free 3-MCPD are to be grouped as sources of 3-MCPD exposure. In animal studies, 3-MCPD is toxic to the kidney and testes and a TDI was previously established by JECFA at 4 µg/kg bw/day using the benchmark dose modelling approach (JECFA 2016) and EFSA is currently revising their TDI (EFSA 2017a).

#### Urinary biomarkers

**Fig. 2:**
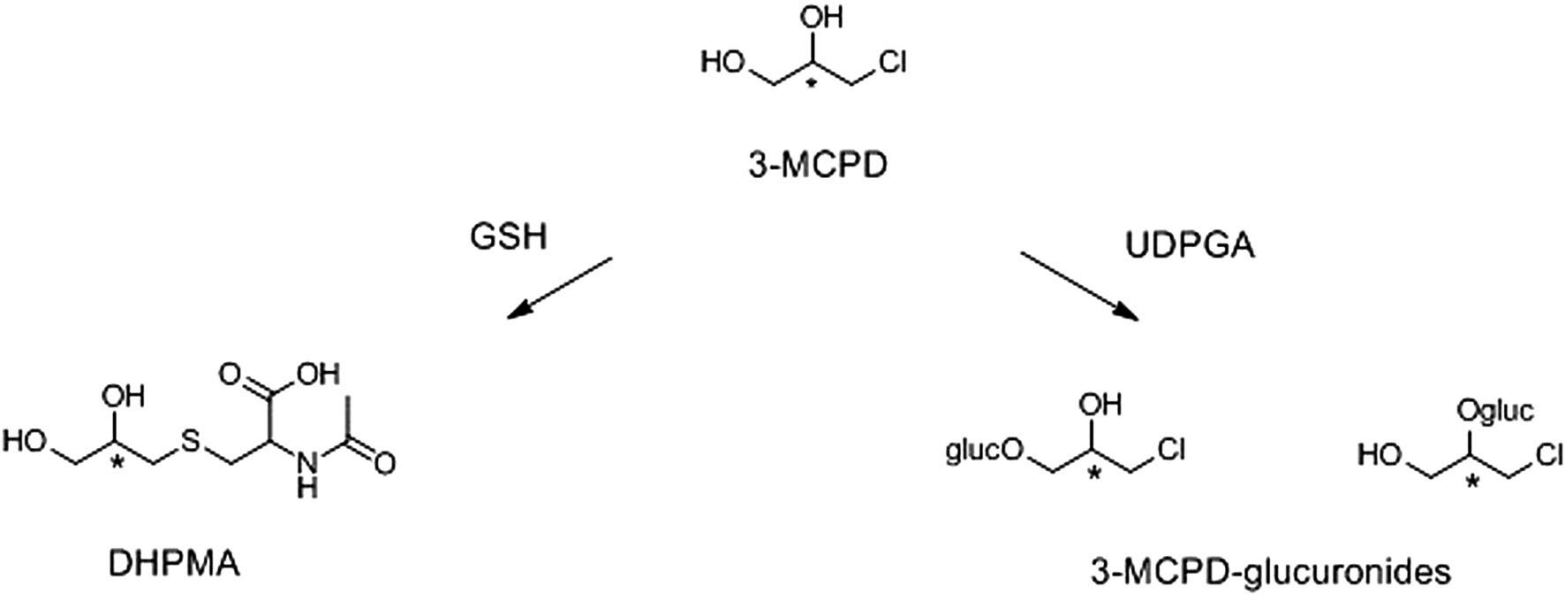
Biomarkers of 3-MCPD exposure including 2,3-dihydroxypropylmercapturic acid (DHPMA) and possible glucuronide conjugates

Two studies in rats, with different methodological approaches, have conducted urinary analysis of 3-MCPD, 3-MCPD dipalmitate, and their metabolites. In one study (Barocelli et al. 2011), urine was analysed for several metabolites (such as the mercapturic acid and glucuronide metabolites), while the other study (Abraham et al. 2013) measured in urine parent 3-MCPD and 3-MCPD dipalmitate. In both studies, a small percentage (< 5%) of the administered 3-MCPD or 3-MCPD dipalmitate was recovered unmetabolized in the urine. Furthermore, the mercapturate of 3-MCPD, 2,3-dihydroxypropylmercapturic acid (DHPMA) (Fig. 2) was present at greater concentrations than 3-MCPD in urine (Barocelli et al. 2011). Glucuronide or sulfo metabolites of 3-MCPD were below the limit of detection (Barocelli et al. 2011).

#### Blood biomarkers

Analysis of blood biomarkers was conducted over 48 h after administration of free 3-MCPD or 3-MCPD dipalmitate (Abraham et al. 2013). The total amount of 3-MCPD was measured using a method that hydrolysed 3-MCPD esters followed by derivatization of all free 3-MCPD. The researchers concluded that overall bioavailability of 3-MCPD and 3-MCPD dipalmitate were similar, with the dipalmitate having 86% of the bioavailability of free 3-MCPD. However, the data also demonstrated that the kinetics of the dipalmitate were delayed relative to free 3-MCPD, with *T*_max_ being reached at 3 h and 22 min, respectively. At the same time, the maximum blood concentration *C*_max_ was 5 times higher for dipalmitate versus free 3-MCPD (0.95 versus 4.85 µg/mL, respectively) (Abraham et al. 2013).

#### Tissue biomarkers

The same study has shown that metabolites of 3-MCPD exposure can be detected in a number of tissues within 24 h after oral exposure: liver, kidney, fat, and intestine (Abraham et al. 2013). As with the analysis in blood, total 3-MCPD in the tissues was measured, accounting for both free and esterified 3-MCPD present. As with circulating metabolites, the total amount of 3-MCPD found in tissues over the first 24 h was similar when free and 3-MCPD dipalmitate were administered orally, but maximum concentrations in the tissues occurred earlier with the free form. This supports the observation with circulating biomarkers of 3-MCPD for more rapid kinetics of the free versus esterified form.

#### Available PBK studies

As described in the previous sections, there have been limited studies conducted analysing the bioavailability and tissue distribution of 3-MCPD after oral exposure. However, so far these data have not been used to develop a pharmacokinetic model for oral exposure to 3-MCPD or its esters.

#### Endogenous formation and accompanying levels of the biomarkers

No sources of endogenous 3-MCPD have been identified, however, some of the urinary metabolites of 3-MCPD, such as the mercapturic acid, are known to be shared with other compounds, such as epichlorohydrin (De Rooij et al. 1997) and glycidol (Eckert et al. 2011). Analytical methods have been developed for 2,3-dihydroxypropyl mercapturic acid (DHPMA), which is a common metabolite of 2-MCPD, 3-MCPD, glycidol and epichlorohydrin, but these methods are unable to differentiate from which parent compound DHMPA is formed (Eckert et al. 2010). Contribution from chemicals like epichlorohydrin to urinary metabolites common to 3-MCPD are unlikely to be significant, except in cases of extreme exposure (De Rooij et al. 1996, 1997), and therefore would only infrequently lead to complication in interpretation of urinary biomarker data. However, using a urinary metabolite like DHPMA as a biomarker for 3-MCPD could be complicated by the presence of co-occurring glycidyl esters (Crews et al. 2013). The hypothesis that urinary DHPMA reflect also a hitherto unknown endogenous C3 metabolite, as previously reported by Eckert et al. (2011), is strongly underpinned by a recent study (Hielscher et al. 2016). In this study, a male volunteer, who avoided intake of food containing refined fats for 2 days, ingested a comparatively high amount of a commercial frying fat with defined levels of 3-MCPD and glycidol fatty acid esters 3 times every 2 days. In parallel DHPMA was determined as urinary biomarker. After oral administration of the frying fat, the average DHMP level remained relatively constant and a clear increase in urinary DHPMA excretion could not be detected. It was concluded that oral exposure to foods with realistic background levels of glycidol or 3-MCPD fatty acid esters is not able to increase the urinary excretion of DHPMA. This study clearly indicates the significance of the formation of this biomarker from another endogenous or exogenous source.

#### Data gaps and research needs

In light of studies that successfully detected 3-MCPD or other markers of 3-MCPD in biological samples (Barocelli et al. 2011; Abraham et al. 2013), significant challenges remain for validating these measurements as biomarkers of dietary exposure to 3-MCPD and its esters. These challenges can be summarized as either technological or biological, but all of these factors contribute to the fact that there are not sufficient data to establish a correlation between the amount of 3-MCPD consumed and any of these biomarkers.

It needs to be determined whether the characterization of the 3-MCPD esters is required, or whether measurement of total 3-MCPD provides enough information to create a correlation. If direct measurement of the esters is necessary to account for the different bioavailability of the esters, then method development would be needed to assess for all esters in blood. This may also require development of broadly available analytical standards for the esters. If urinary analysis is pursued, there would be the need for additional analytical standards for many of the urinary metabolites, such as the glucuronide- and sulfo-conjugates (Barocelli et al. 2011).

Biologically, validation of biomarkers for exposure needs to account for the complexity of differences in bioavailability between both free and esterified 3-MCPD, as well as between the different 3-MCPD esters. Currently, only the dipalmitate has been evaluated (Abraham et al. 2013). As evidenced by these data, there were differences between bioavailability of the free and esterified forms. However, due to the rapid hydrolysis of esters in the body (Abraham et al. 2013; EFSA 2016), development of biomarkers for the different 3-MCPD esters is likely unnecessary.

### Glycidyl esters

#### Characterization, formation, occurrence and public health concern

2,3-Epoxy-1-propanol (glycidol) esters were originally used as raw materials and stabilizers to produce polymers in the cosmetic and pharmaceutical industries. In 2009, the German Federal Institute for Risk Assessment (BfR) detected glycidyl fatty acid esters (GEs) in refined vegetable fats and expressed concern over the possible release of the suspected human carcinogen glycidol during gastrointestinal digestion (BfR 2009a, b; Bakhiya et al. 2011). Subsequently, GEs were discovered in refined edible oils and fats or foods containing them (Masukawa et al. 2010; Weißhaar and Perz 2010; Blumhorst et al. 2011, 2013; Kuhlmann 2011; Craft et al. 2013; Crews et al. 2013; MacMahon et al. 2013; Wöhrlin et al. 2015). Studies indicate that heat treatment generates GEs and, particularly, the deodorization process in oil refining (Franke et al. 2009; Matthäus et al. 2011). Recently, Inagaki et al. (2016) demonstrated high concentrations of GEs in meat samples heated at high temperatures. The composition of the heat-formed GEs agreed with the fatty acid composition of non-heated samples, indicating that the fatty acids and triglycerides in foods could be converted to GEs.

Carcinogenicity evaluations reported that subcutaneous injection of glycidyl oleate or stearate in mice resulted in low or insignificant increases in local tumour incidences (Walpole 1958; Swern et al. 1970; Van Duuren et al. 1972). Thus, the IARC classified glycidyl oleate and stearate as “Not classifiable as to its carcinogenicity to humans” (IARC 1976, 1987). Genotoxicity studies of glycidyl linoleate, including the Ames test in five strains, chromosome aberration test in Chinese hamster lung cells, and micronucleus formation in mice, suggested that glycidyl linoleate is to be considered non-genotoxic, since a positive result in the Ames test of glycidyl linoleate was attributed to the release of glycidol (Ikeda et al. 2012). Glycidol has shown mutagenic effects in vitro in bacterial tests with and without metabolic activation, and a wide spectrum of genotoxic effects in mammalian cell systems (genetic mutations, chromosomal aberrations, sister chromatid exchanges, unscheduled DNA synthesis). In vivo genotoxicity tests were less conclusive, some positive results were reported in micronucleus assays in mice after intraperitoneal administration, but not in others (IARC 2000; EFSA 2016). The toxicological and carcinogenic potential of glycidol has sufficiently been investigated by NTP and IARC (NTP 1990; IARC 2000). Following evidence of carcinogenicity in mice and rats, the IARC evaluated glycidol as “probably carcinogenic to humans” (Group 2A), although no epidemiological data were available.

#### Urinary biomarkers

**Fig. 3:**
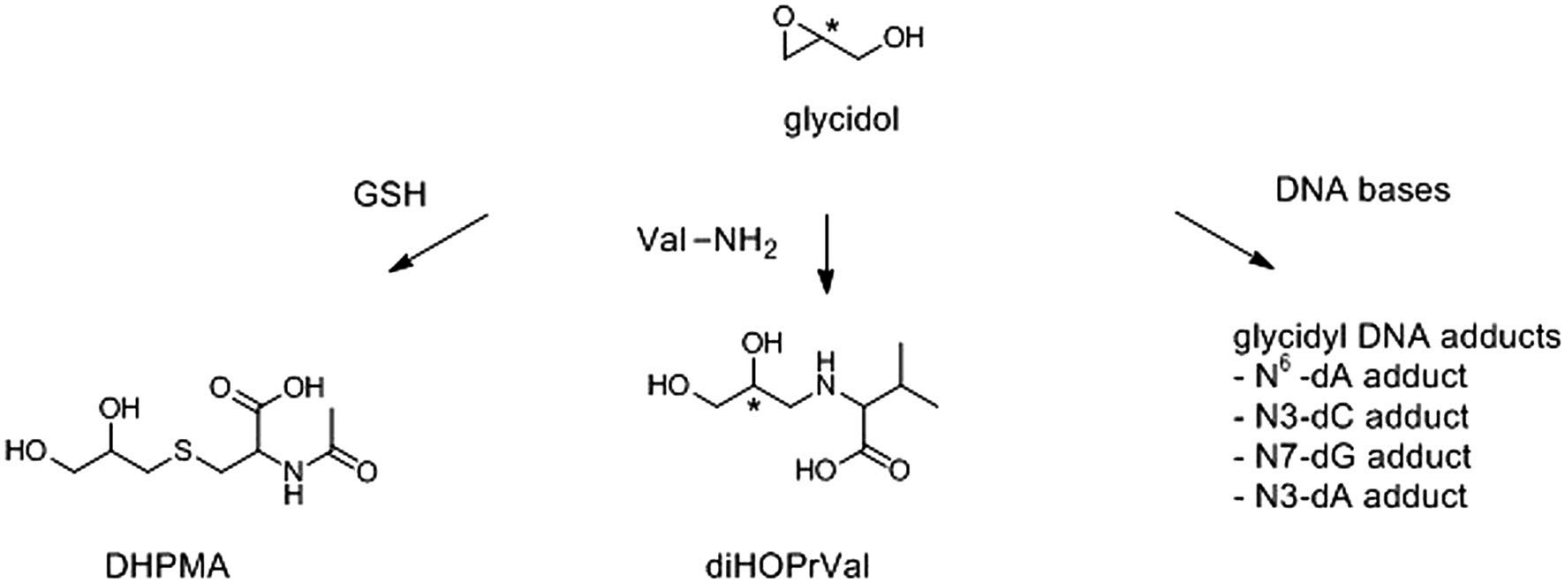
Biomarkers of glycidol/glycidylester exposure including 2,3-dihydroxypropylmercapturic acid (DHPMA) and *N*-(2,3-dihydroxypropyl)valine (diOHPrVal) and several DNA adducts

2,3-Dihydroxypropyl mercapturic acid (DHPMA), previously discussed in regard to 3-MCPD (Sect. 2.2.2), is also a urinary biomarker of glycidol and its esters (Fig. 3). In addition to the studies of DHMPA described above (Eckert et al. 2011), Appel et al. (2013) also measured DHPMA to investigate the oral bioavailability of glycidol from GEs in rats. Rats were administered glycidyl palmitate or an equimolar dose of glycidol and urine was collected at 0–8, 8–24, and 24–48 h after dosing. The urinary DHPMA level reached the highest concentration at 0–8 h, and almost all the DHPMA was excreted at 8–24 h in both treatment groups. No significant differences occurred between the glycidol and glycidyl palmitate groups.

#### Blood biomarkers

Glycidyl-haemoglobin adducts have been used to assess GEs because GEs are mainly absorbed as glycidol (Wakabayashi et al. 2012; Appel et al. 2013). Landin et al. (1996) demonstrated the covalent binding of glycidol to N-terminal valine of haemoglobin in vitro. Furthermore, the *N*-(2,3-dihydroxypropyl)valine (diHOPrVal) adduct (Fig. 3) was detected using the *N-*alkyl Edman GC-MS/MS method (Landin et al. 1996), initially established to investigate external and/or internal exposure to epichlorohydrin. Background levels of diHOPrVal were detected in control subjects (Landin et al. 1996, 1997). More recently, Aasa et al. (2017) developed a more convenient analytical method to evaluate diHOPrVal using LC-MS/MS and the new derivatizing reagent, fluorescein isothiocyanate, as an alternative to earlier GC-MS/MS methods. They confirmed correlations among administered dose of glycidol, diHOPrVal levels, and micronuclei frequencies in experiments on mice. Furthermore, Hielscher et al. (2017) reported a similar method using fluorescein isothiocyanate and UPLC-MS/MS technique. Glycidol forms dihydroxypropyl adducts in biomolecules. Related substances in foodstuffs (including anhydrosugars, allyl alcohol, and glycerol halohydrins), have been discussed as possible precursors of glycidol (Hauschild and Petit 1956; Jones 1975; Patel et al. 1980; Piasecki et al. 1990; Hamlet 1998; Ishidao et al. 2002) that might account for background diHOPrVal. Furthermore, subsequent studies revealed that diHOPrVal levels in tobacco smokers are higher than those in non-smokers (Landin et al. 1996), and levels in rats fed a fried diet were higher than those in control rats (Landin et al. 2000). Moreover, Honda et al. (2011, 2012) used diHOPrVal for exposure evaluation in humans who ingested diacylglycerol (DAG) oil containing high levels of GEs compared to other edible oils (Masukawa et al. 2010). The diHOPrVal was detected in all subjects regardless of DAG exposure level, and did not differ between DAG- and non-exposed subjects. GE-induced internal exposure to glycidol was possibly not markedly higher than that induced by other unknown sources of internal exposure, which could contribute to background diHOPrVal levels.

Furthermore, diHOPrVal formation by glycidol exposure had only been demonstrated in vitro and had not been characterized as an in vivo exposure marker of glycidol and GEs until the early 2000s, whereas the diHOPrVal formed by epichlorohydrin exposure in rats had been (Landin et al. 1999). Following this situation, Appel et al. (2013) demonstrated diHOPrVal formation in rats after oral administration of glycidol and glycidyl palmitate. Formation was slightly delayed in the glycidyl palmitate group while total levels were comparable in both groups, indicating that glycidyl palmitate was entirely hydrolyzed to glycidol and palmitic acid. Moreover, Honda et al. (2014) confirmed the dose-dependent formation, chemical stability in vivo, and sensitivity of diHOPrVal measurements in kinetics studies in rats orally administered glycidol. Moreover, in vivo doses calculated from the second-order reaction rate constant in vitro agreed with the area under the concentration–time curve (AUC) values determined in rat short-term toxicokinetic studies (Wakabayashi et al. 2012). Therefore, diHOPrVal is considered useful for estimating AUC values, at least following controlled short-term oral exposure. DiHOPrVal is also of use to estimate daily exposure levels from accumulated or steady-state levels using mathematical models (Fennell et al. 1992; Granath et al. 1992) because diHOPrVal would be stable in vivo over the lifetime of the erythrocyte (Honda et al. 2014).

#### Tissue biomarkers

In vitro studies demonstrated that glycidol reacts with DNA bases and, therefore, glycidyl-DNA adducts could be tissue biomarkers of glycidol-related compounds including GEs, which release glycidol during gastrointestinal digestion. Hemminki et al. (1980) reported that glycidol reacted with deoxyguanosine to produce 1,7-(or 1,9)-dialkylguanine, with deoxyadenosine to produce adducts at N6, with deoxycytidine at N3 by incubating with each deoxyribonucleoside. Subsequently, Segal et al. (1990) identified a thymidine and uridine adduct at N3 formed by the in vitro reaction of glycidol and calf thymus DNA. They also demonstrated that N3 uridine adducts were rapidly formed by hydrolytic deamination of N3 cytosine adducts. Moreover, Solomon (1998) reported that several epoxides including glycidol formed adducts with deoxyguanosine at N7 and with deoxyadenosine at N3 as major adducts. Toshima et al. (2003) also reported that glycidol selectively alkylated DNA at the N7 sites of the guanines. Furthermore, Ozcagli et al. (2016) reported high purine base damage, indicating N7 guanine alkylation in glycidol-induced HEK-293 cells. The N7 of guanine is the major site of alkylation by epoxides that may lead eventually to point mutations by depurination (Melnick 2002). The glycidol-DNA adduct formation in vivo has not been investigated. However, these findings indicate that DNA adducts may be formed by exposure to glycidol in vitro and in vivo, and could be a biomarker of biologically effective doses of glycidol released from GEs in target tissues and organs.

#### Available PBK studies

No PBK model for GEs has been developed to date. In related studies, Frank et al. (2013) investigated the fate of GEs including their biotransformation into glycidol. Static and dynamic gastrointestinal models were used to determine lipase and pH effects on different GEs. Although GEs are stable at pH 1.7–4.8, they are rapidly hydrolysed following the addition of lipase. Under the conditions of dynamic simulation and in food matrix models, the transformation to glycerol was very small. Transformation to MCPD occurred only after extended incubation time and at very small quantities. These results agreed with those of rat toxicokinetic and bioavailability studies (Wakabayashi et al. 2012; Appel et al. 2013). In contrast, Onami et al. (2015) pointed out that glycidol or GEs might be converted to 3-MCPD in the rat gastrointestinal tract based on their in vivo experiments (single oral gavage, 1 time point) and ex vivo experiments (incubation with gut content samples). These studies did not derive PBK parameter values directly. However, toxicokinetic information reported in these studies might be useful for constructing PBK models.

#### Endogenous formation and accompanying levels of available biomarkers

Eckert et al. (2011) reported relatively high background DHPMA levels in human urine, which strongly correlated with urinary creatinine (*r* = 0.945, *p* < 0.0001, regression analysis). Similarly, Andreoli et al. (2015) reported a weak correlation between DHPMA and creatinine in human urine (*r* = 0.176, *p* < 0.022, in Spearman’s correlation); however, the correlation was not as high as that observed by Eckert et al. (2011). Whether the difference between both studies is only attributable to the difference in analytical methods is unknown. Moreover, the smoking status of the human subjects, which might affect the external exposure to glycidol, was not correlated with urinary DHPMA concentrations (Eckert et al. 2011; Andreoli et al. 2015). Thus, these findings raised the hypothesis that an endogenous bioprocess could generate DHPMA. The correlation of DHPMA levels with 3-hydroxypropyl mercapturic acid (3-HPMA), an acrolein biomarker, indicates an identical C3 precursor.

#### Data gaps and research needs

Extending the applicability of DHPMA to GE exposure evaluation will require establishment of dose-dependency and highly sensitive analytical methodology because GE food levels are normally rather low. Furthermore, DHPMA is not specific to glycidol because it is derivable from glycidol and 3-MCPD (Jones 1975). The relationship between glycidol exposure and DHPMA levels has not been confirmed in humans. Therefore, the combination analysis of DHPMA and haemoglobin adducts, which are not considered 3-MCPD biomarkers may lead to more precise GE exposure evaluation in humans, covering short- and long-term exposures.

Haemoglobin adducts, including diHOPrVal, cannot be used as tissue biomarkers for assessing target organ or tissue exposure levels. Therefore, for cancer risk assessment, tissue biomarkers such as DNA adducts are considered superior to haemoglobin adducts in target tissues. However, contrary to mercapturic acids, haemoglobin adducts would be more suitable as chronic exposure biomarkers. Further studies on background levels of diHOPrVal in humans and animals and the use of other protein adducts as biomarkers are required.

No in vivo studies are available on glycidol-DNA adduct formation. Therefore, the value of glycidyl-DNA adducts to determine exposure in humans who ingest foods containing small amounts of GEs needs clarification. The elucidation of DNA adduct types formed and their levels in animal experiments would be required. Clearly, large-scale sampling of tissue DNA adducts is difficult and would only be applicable in limited studies/population groups.

### Furan

#### Characterization, formation, occurrence and public health concern

**Fig. 4:**
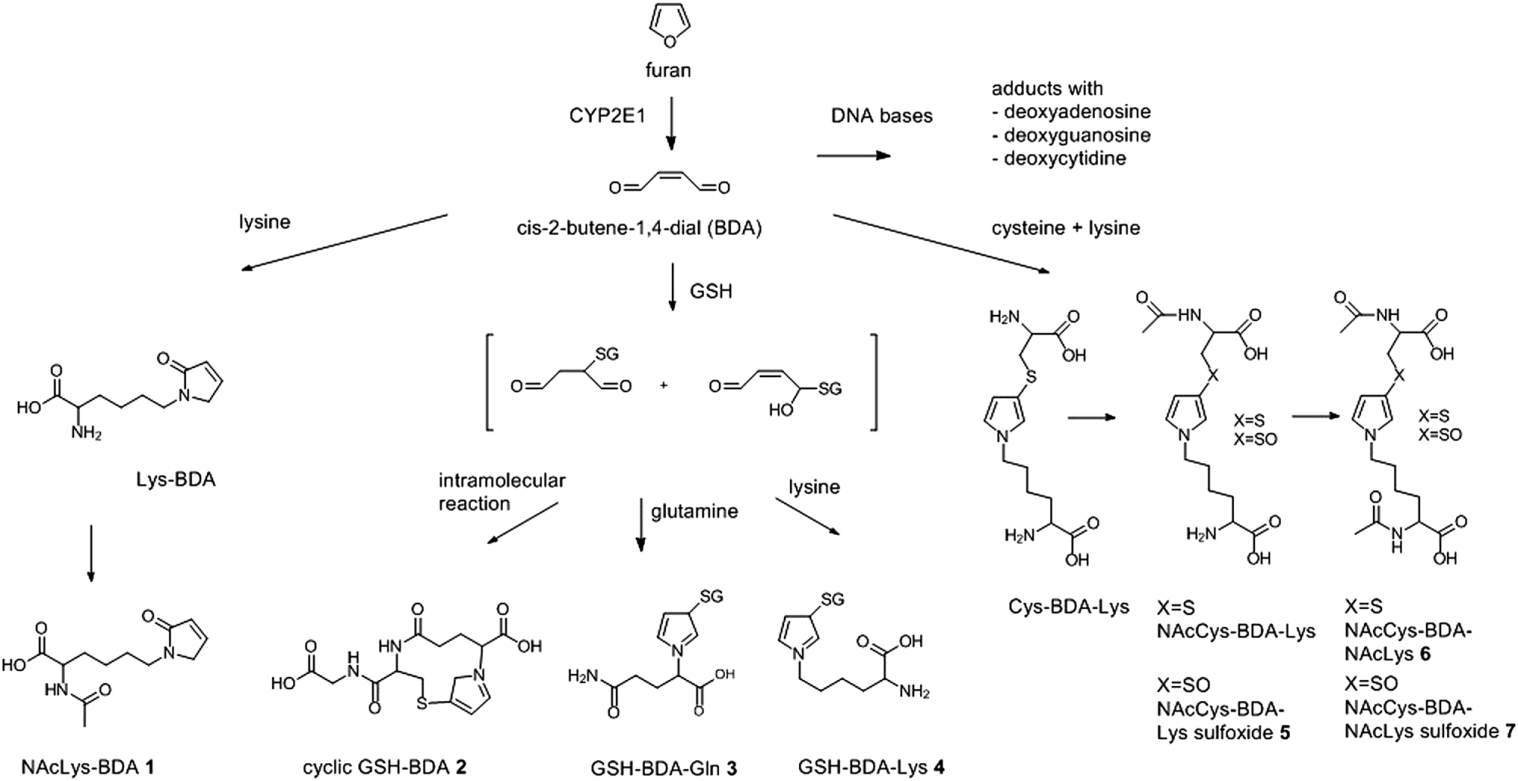
Metabolic pathways of furan leading to formation of potential biomarkers of furan exposure. Metabolites identified as possible biomarkers of exposure in rat urine include: NAcLys-BDA (1), which results from the reaction of BDA with lysine and subsequent *N*-acetylation; a mono GSH-BDA conjugate (2), which results from conjugation with GSH and intermolecular reaction with the α-amino group of the GSH glutamyl residue; metabolites in which GSH is crosslinked by BDA with glutamic acid (3) or lysine (4); NAcCys-BDA-NAcLys (6) and its sulfoxide (7), which represent *N*-acetylated crosslinks of cysteine and lysine by BDA. In humans, NAcCys-BDA-Lys sulfoxide (5) was identified as a potential urinary biomarker of human exposure to furan. Metabolites in which cysteine and lysine are cross-linked by BDA may result from either degradation of protein adducts or enzymatic processing of the GSH-BDA-Lys conjugate (4). Note that conjugation of BDA with GSH can also occur at the 2-position instead of the 3-position of the pyrrole ring. For reasons of clarity, only the 3-substituted metabolites are shown

Furan is a highly volatile, oxygen containing heterocyclic compound that was found to be formed unintentionally in heat processed foods, particularly in coffee and in canned and jarred foods. Multiple precursors have been identified that are natural food components, including carbohydrates, amino acids, polyunsaturated fatty acids and carotenoids. Different precursors and formation pathways have been discussed for dry heating (e.g. coffee roasting) or wet heating (retort sterilization) (Mariotti et al. 2013). Retort processing prevents furan from evaporating, and during coffee roasting the furan formed remains ‘trapped’ inside the roasted bean and even in roast and ground coffee. Once ‘bound’ in the matrix furan does not easily evaporate, for example upon re-heating of jarred foods prior to consumption (Goldmann et al. 2005; Roberts et al. 2008; Kim et al. 2009) or after brewing of coffee where a considerable amount may remain in the brew (Goldmann et al. 2005; Guenther et al. 2010). The final concentrations of furan depend on multiple factors such as industrial or home processing, preparation for consumption (type of brewing, microwave or stove heating) and individual consumption habits as, e.g. stirring. Thus analytical data generated on products as taken from the shelf do not reflect the amounts ingested by the consumer. Furthermore, reliable quantitative information on evaporation losses are unavailable and difficult to estimate or generalize. Although furan can be found in the environment (combustion, cigarette smoke, etc.), main human exposure was identified to occur via food (JECFA 2011). In rodent studies, furan is toxic to the liver and a carcinogen (NTP 1993; JECFA 2011; Von Tungeln et al. 2017). There is sufficient evidence that toxicity is induced by cytochrome P450 (CYP 2E1) mediated bioactivation to a reactive metabolite, *cis*-2-butene-1,4-dial (BDA) (Fig. 4) that is able to covalently bind to macromolecules such as proteins and DNA. There is some controversy about the genotoxicity of furan due to inconclusive results of in vivo and in vitro genotoxicity tests. Some scientists argue for a non-genotoxic mechanism of action secondary to cytotoxicity exerted in the liver (Ding et al. 2012; McDaniel et al. 2012; Terrell et al. 2014; Churchwell et al. 2015), which is also supported by gene expression studies (Chen et al. 2010, 2012; Dong et al. 2016, Tryndvak et al. 2017). However, JECFA concluded that in the carcinogenicity of furan a genotoxic mechanism of action cannot be excluded and a non-threshold effect was assumed. Application of the Margin of Exposure (MOE) approach indicated a potential human health concern and it was recommended to explore possible measures that could reduce consumer exposure (JECFA 2011). In a recent evaluation of the risks for public health related to the presence of furan in food, the EFSA Panel on Contaminants in the Food Chain concluded that there was limited evidence for a direct genotoxic mechanism in furan carcinogenicity, whereas there was clear evidence for an involvement of indirect mechanisms, including epigenetic changes, inflammation, oxidative DNA damage and regenerative cell proliferation subsequent to liver injury (EFSA 2017b). The Panel also noted that the contribution of these factors to furan carcinogenicity may well vary with dose, duration of exposure and severity of tissue damage (EFSA 2017b). However, considering the uncertainties regarding involvement of direct genotoxic mechanisms in furan carcinogenicity, the Panel decided that a tolerable daily intake (TDI) could not be established and applied the MOE approach to the risk characterization using both non-neoplastic and neoplastic effects. The Panel concluded that the calculated MOEs, which were below 100 for non-neoplastic effects and smaller than 10,000 for neoplastic effects, indicate a health concern (EFSA 2017b).

More recently, occurrence and toxicological information have become available on alkylated furans, particularly 2- and 3-methylfuran, and 2,5-dimethylfuran that provide some evidence for similar (or overlapping) formation pathways and co-occurrence, though occurrence does not always seem to be correlated (e.g. Becalski et al. 2010; Chaichi et al. 2015). Occurrence, absorption, distribution, metabolism, and excretion (ADME) and toxicological information is still limited on these compounds and they are not addressed in this study. In its recent assessment, the European Food Safety Authority Panel on Contaminants in the Food Chain considered that a full assessment of methylfurans is presently not possible due to a lack of toxicity and occurrence data. Nevertheless, it was considered adequate to group these compounds and dose additivity was assumed (EFSA 2017a).

#### Urinary biomarkers

The ADME characteristics of furan after oral intake in animal studies were extensively reviewed by JECFA (2011). Important routes of furan elimination are expired air (CO_2_ and other volatiles), urine and faeces, accounting for elimination of 40, 20 and 22% of the administered dose, respectively, 24 h after a single dose of 8 mg/kg bw (Burka et al. 1991). A number of metabolites of furan were isolated from the urine after oral administration of radiolabelled furan to rats (Burka et al. 1991). They were suspected to be glutathione (GSH) and mercapturic acid conjugates derived from the reactive dialdehyde intermediate of furan, *cis*-2-butene-1,4-dial (BDA), generated by CYP2E1 activity in the liver (Fig. 4), but the structures were initially not identified (Burka et al. 1991). The identity of urinary metabolites was more recently studied in furan treated rats. Of several conjugates derived from the reaction of furan with GSH (as identified after reaction of BDA (e.g. after hydrolysis of the BDA precursor 2,5-diacetoxy-2,5-dihydrofuran) with GSH in vitro or in microsomal extracts), only the mono-glutathione conjugate (GSH-BDA) (**2**) (Fig. 4) was identified in rat urine after oral dosing (Peterson 2006; Kellert et al. 2008). This GSH-BDA adduct was proposed to be a promising urinary biomarker. Additional metabolites generated from the reaction of BDA or GSH-BDA with free or protein-bound lysine as well as BDA-derived cysteine-lysine crosslinks were identified in rat urine after oral furan treatment in vivo (Lu et al. 2009). These include R-2-acetylamino-6-(2,5-dihydro-2-oxo-1H-pyrrol-1-yl)-1-hexanoic acid, which results from the reaction of BDA with lysine and subsequent *N*-acetylation (NAcLys-BDA) (**1**) (Fig. 4), as well as *N*-acetyl-*S*-[1-(5-acetylamino-5-carboxypentyl)-1*H*-pyrrol-3-yl]-l-cysteine (NAcCys-BDA-NAcLys) (**6**) and its sulfoxide (**7**) (Fig. 4), which represent *N*-acetylated crosslinks of cysteine and lysine by BDA (Lu et al. 2009). In a mass spectrometric analysis of urine from furan treated rats, combined with multivariate analysis for metabolic profiling, a number of metabolites were identified as possible furan biomarkers of exposure. These were characterized as lysine- (**1**), mercapturate- (**6**) or mercapturate sulfoxide (**7**) conjugates, a GSH and a glutamic acid adduct (**3**), all derived from the BDA metabolite and containing a pyrrole ring structure derived from the ring closure of the BDA molecule (Kellert et al. 2008) (Fig. 4), and overlapping with the ones identified by Peterson and Lu above. No examples of application of these biomarkers to correlate dietary furan exposure in animals or humans are available yet. However, one study addressed the presence of urinary furan exposure biomarkers in three cohorts of smokers versus non-smokers comprising between 5 and 15 subjects per group (Grill et al. 2015). Metabolites derived from direct interaction of BDA with lysine and from crosslinks of cysteine-BDA-lysine (Fig. 4) were analysed but not all metabolites were consistently identified and quantified due to interfering peaks or high variability of the results in the LC-MS/MS analysis. While the NAcCys-BDA-NAcLys (**6**) and its sulfoxide (**7**), consistently reported to occur in rat urine, were below the limit of detection in humans pointing at a possible interspecies difference, one of the *N*-acetylcysteine-BDA-lysine metabolites, the NAcCys-BDA-Lys sulfoxide metabolite (**5**) (Fig. 4), was strongly correlated with smoking in all three cohort studies from the US, Shanghai, and Singapore. Its levels were more than 10 times higher in urine from smokers than in urine from non-smokers, and levels decreased immediately upon cessation of smoking. The NAcCys-BDA-Lys sulfoxide (**5**) was postulated to be a degradation product of adducted proteins, and as such a promising marker for furan metabolism to BDA and also a potential biomarker of furan toxicity (furan induced protein damage) (Grill et al. 2015). Besides degradation of protein adducts, enzymatic processing of the GSH-BDA-Lys conjugate (**4**) (Fig. 4) may also lead to the formation of metabolites in which cysteine and lysine are cross-linked by BDA.

#### Blood biomarkers

Twenty-four hours after oral administration of radioactively labelled furan to rats, less than 1% (0.42%) of the dose was present in blood (Burka et al. 1991). While radioactivity was eliminated quickly from liver, elimination from kidney and blood was more slowly, with levels in blood remaining rather constant for 8 days following a single dose treatment, indicating some covalent binding to macromolecules. The level of binding increased with repeated application compared to single dosing over 8 days. In a recent toxicokinetic study in rats with single oral furan administration at a dose of 0.92 mg/kg bw/day, furan levels in blood decreased rapidly with a half-life of 1.3 h and were undetectable 8 h after dosing (Churchwell et al. 2015). No blood-specific macromolecular adducts with furan were characterized and no studies related to blood exposure markers were found in the published literature.

#### Tissue biomarkers

Studies on the identification of tissue macromolecular adducts have focused on the liver, since it is the organ of bio-activation to BDA, and the organ with the highest furan concentration in rodent ADME studies with oral administration of radioactively labelled furan. Due to the non-extractability of furan-associated radioactivity, it was suggested that furan was bound to proteins in rat liver. With repeated dosing at daily doses of 8 mg/kg for 8 days, furan levels increased almost linearly up to day 4, reaching a plateau between 4 and 8 days of dosing. No DNA binding was initially identified in rat liver in vivo (Burka et al. 1991), whereas DNA adducts with deoxyadenosine, deoxycytidine and deoxyguanosine were identified after reaction of BDA with calf thymus DNA or DNA isolated from *S. typhimurium* strain TA104 after treatment with mutagenic concentrations of BDA (Byrns et al. 2006). In a more recent study employing accelerator mass spectrometry (AMS), a significant, dose-related increase in ^14^C-content in rat liver DNA (corresponding to 1.7 ± 0.7 and 32.5 ± 21.2 adducts/10^8^ nucleotides at 0.1 and 2.0 mg/kg bw, respectively) was observed after oral administration of [3,4-^14^C]-furan (0.1 and 2.0 mg/kg bw) to F344 rats. However, DNA adducts with deoxyadenosine, deoxycytidine and deoxyguanosine identified after reaction of BDA with DNA in vitro were not detected in rat liver using LC-MS/MS, even after treatment with a single high dose (30 mg/kg bw) of furan or after repeated administration of lower doses (2 mg/kg bw/day) for 28 days, with LODs of 3.3–6.6 adducts/10^8^ nucleotides (Neuwirth et al. 2012). Although radioactivity was significantly and covalently bound to DNA and metabolic incorporation of ^14^C was excluded, attempts to characterize the DNA modifications carrying the radiolabel failed (Neuwirth et al. 2012). It was suggested that the primary DNA adducts may rearrange further, possibly also forming crosslinks that are structurally difficult to assess. The formation of crosslinks has been proposed indirectly based on results from a Comet assay in CHO cells and turkey foetal liver cells (Marinari et al. 1984; Jeffrey et al. 2012), but no chemical structural characterization was provided.

In line with the study by Neuwirth et al. (2012), no evidence for DNA adduct formation (BDA-2′-deoxycytidine) in rat liver was found by Churchwell et al. (2015) using LC-MS/MS after dosing for up to 360 days at a dose of 9.2 mg/kg bw/day.

While several proteins were recently identified as targets for covalent modification by furan reactive metabolites, chemical characterization of the adducted proteins is still lacking (Moro et al. 2012). Recently, Nunes et al. (2016) reported formation of a cross-link between GSH-BDA and lysine 107 of histone H2B in livers of male F344 rats treated with furan. This modification was detected in vivo after treatment with furan (0.92 mg/kg bw) and was observed prior to the occurrence of epigenetic histone alterations, leading the authors to conclude that covalent histone modification by reactive furan metabolites may be linked to initial stages of tumour development by furan. In addition to providing mechanistic insight into furan carcinogenicity, the identified cross-link between GSH-BDA and histone H2B may serve as a biomarker of furan exposure.

#### Available PBK studies

Based on inhalation studies in F344 rats, a physiologically based pharmacokinetic (PBK) model was developed for furan. The model accurately predicted blood and liver concentrations of rats exposed to constant furan inhalation, which was validated with independent inhalation studies. Model simulations of furan metabolism in the rat after a single oral dose of 8 mg/kg bw predicted metabolism of 84% of the dose and 16% exhaled as the parent compound (Kedderis et al. 1993), which is in good agreement with data reported by Burka et al. (1991) who determined 86% of the dose metabolized and 14% of the dose exhaled 24 h after a single 8 mg/kg dose. Furan biotransformation kinetic parameters determined with freshly isolated rat hepatocytes in vitro were found to accurately predict furan pharmacokinetics in vivo in rats (Kedderis et al. 1993). Kinetic parameters obtained from mouse and human primary hepatocytes were furthermore used to build species-specific PBK models for furan inhalation exposure that revealed important interspecies differences with respect to pulmonary absorption, steady-state blood concentrations of furan and liver exposure to the metabolite BDA (Kedderis and Held 1996). No PBK model studies are available to date on oral (dietary) exposure to furan in humans, or on other metabolic reactions apart from oxidation to BDA, such as, e.g. glutathione conjugation.

#### Endogenous formation and accompanying levels of the biomarkers

Of several potential biomarkers of furan exposure identified by metabolic profiling of rat urine, three metabolites, i.e. the NAcLys-BDA adduct (**1**), the NAcLys-BDA-NAcCys (**6**) as well as an unidentified metabolite, were also found to be present in urine of control animals (Kellert et al. 2008). Although quantitative analysis was not performed, the ratio between these three metabolites in treated versus control animals varied considerably. It is unclear if this background may be due to low level exposure of control animals to furan via rodent diet or endogenous formation unrelated to furan exposure (Kellert et al. 2008). There is evidence that chemical oxidation of deoxyribose in DNA gives rise to the *trans*-isomer of 2-butene-1,4-dial (Chen et al. 2004). While this may present a plausible pathway for endogenous formation of BDA adducts, there are at present no data to confirm formation of these metabolites in vitro or in vivo. In summary, endogenous formation cannot be excluded to date, but evidence is lacking to back this possibility.

#### Data gaps and research needs

The application of urinary biomarkers to monitor human exposure to furan has not been investigated yet, including the use of specific urinary markers as representatives of overall furan exposure or specifically for dietary furan exposure. Aspects that need further investigations include improved analytical methods for sensitive analysis of potential urinary biomarkers of furan exposure (e.g. NAcLys-BDA (**1**), the mono-glutathione conjugate GSH-BDA (**2**) or the NAcCys-BDA-Lys sulfoxide (**5**)) at expected low dose dietary exposure levels as well as data on the correlation of levels of different urinary exposure biomarkers to support a single biomarker approach. Besides urinary biomarkers, the suitability of haemoglobin adducts as a potential avenue to monitor chronic low dose exposure to furan should be explored. Furthermore, there is a need to clarify potential endogenous formation of furan and 2-butene-1,4-dial, e.g. by using duplicate diet methodology. To this end it should be emphasized that duplicate diet sampling needs to reflect the meal consumption process to take account of the volatility of furan.

### Acrolein

#### Characterization, formation, occurrence and public health concern

Acrolein is the most reactive of α,β-unsaturated aldehydes that is formed for instance upon heating of cooking oil, and also detected as a constituent of cigarette smoke (lARC 1993; Kehrer and Biswal 2000; ATSDR 2007; Tang et al. 2011; Guillén and Uriarte 2012; Moghe et al. 2015). Acrolein has also been reported to be a product formed during fermentation and ripening processes (Gomes et al. 2002) and to be generated endogenously during cellular lipid peroxidation and other processes (Uchida et al. 1998; Stevens and Maier 2008). In addition, acrolein is well known to be formed from several other structurally related xenobiotica and as a metabolic by-product from some drugs such as the oxazaphosphorine cytostatics (Burcham 2017). Being an α,β-unsaturated aldehyde, acrolein reacts with cellular nucleophiles including proteins and DNA and forms conjugates with glutathione. The reaction with glutathione presents an important pathway for its detoxification unless glutathione levels are depleted (Ohno et al. 1985; Silva and O’Brien 1989; Kehrer and Biswal 2000; Kiwamoto et al. 2015).

#### Urinary biomarkers

**Fig. 5:**
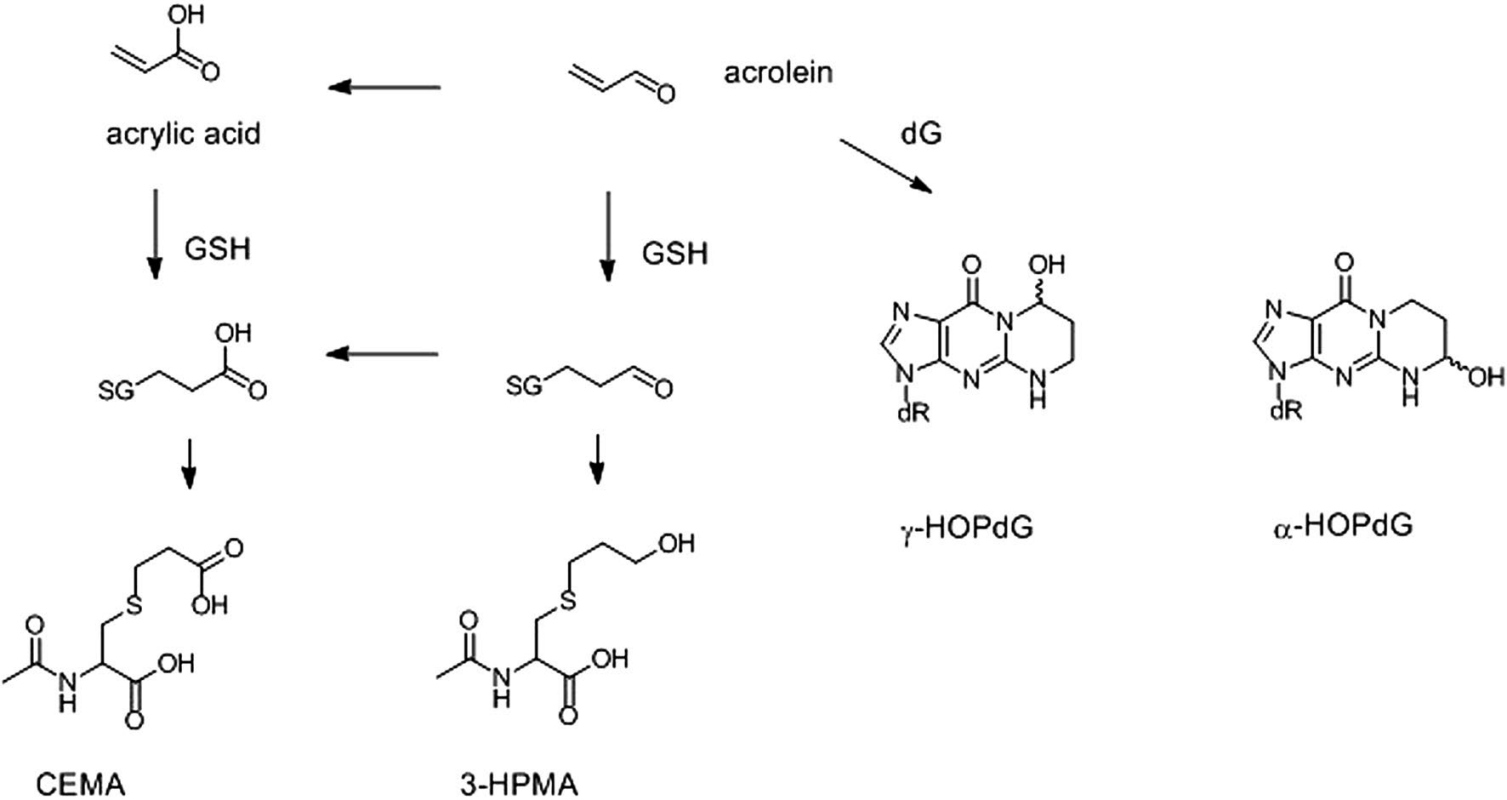
Biomarkers of acrolein exposure including 3-hydroxypropylmercapturic acid (3-HPMA), 2-carboxyethylmercapturic acid (CEMA), gamma-hydroxy-1,*N*^2^-propano-2′-deoxyguanosine (γ-HOPdG) and alpha-hydroxy-1,*N*^2^-propano-2′-deoxyguanosine (α-HOPdG)

An important urinary biomarker reflecting exposure to acrolein is 3-hydroxypropylmercapturic acid (3-HPMA) (Fig. 5), a major urinary metabolite of acrolein formed from the glutathione conjugate of acrolein (ATSDR 2007; Stevens and Maier 2008). Urinary 3-HPMA has been used as a biomarker for acrolein exposure in large- and smaller-scale biomonitoring studies with healthy non-smoking human volunteers (Carmella et al. 2007; Schettgen et al. 2008; Alwis et al. 2015; Hecht et al. 2015; Park et al. 2015). The DFG-Senate Commission on Food Safety (SKLM) concluded that 3-HPMA can be used as biomarker of exposure over especially the last 48 h (Guth et al. 2013). In a pilot study monitoring the mercapturic acid content of spot urine samples from 14 occupationally non-exposed non-smokers the excretion of 3-HPMA was even found to be at least three times that of *N*-acetyl-*S*-(2-carbamoylethyl)-l-cysteine (AAMA), a biomarker of exposure to acrylamide (Schettgen et al. 2008). A subsequent study in 13 human volunteers showed similar results, since upon consumption of a test meal of potato crisps/chips experimentally heat treated to achieve a high acrylamide intake (1 mg per person), the excretion of 3-HPMA was found to be substantially (15-fold) higher than that of the acrylamide-related mercapturic acids (Watzek et al. 2012a).

Another urinary metabolite of acrolein is 2-carboxyethylmercapturic acid (CEMA) (*N*-acetyl-*S*-2-carboxyethylcysteine) formed via either oxidation of acrolein to acrylic acid and subsequent glutathione conjugation or by oxidation of the glutathione conjugate of acrolein, ultimately both leading to formation of CEMA (Fig. 5) (Kaye 1973; Linhart et al. 1996; Alwis et al. 2015).

When using 3-HPMA as a urinary biomarker for acrolein exposure it is of importance to take into account that several allylic compounds may also metabolize to 3-HPMA, including allylamine, allyl halides, and allyl alcohol and ester (ATSDR 2007).

#### Blood biomarkers

Acrolein reacts with deoxyguanosine to form 1,*N*^*2*^-propanodeoxyguanosine adducts which were chemically characterized after in vitro incubations with calf thymus DNA (Chung et al. 1984). The major adducts are thought to be formed from Michael-type addition of *N*^*2*^-amine of deoxyguanosine (dG) to C3 of acrolein, giving stereoisomeric gamma-hydroxypropanodeoxyguanosine (γ-HOPdG) (Fig. 5). The minor adducts are formed from Michael addition of N1 of dG to the C3 position, producing stereoisomeric alpha-hydroxy-1,*N*^2^-propano-2′-deoxyguanosine (α-HOPdG) (Chung et al. 1984) (Fig. 5).

These two isomers of 1,*N*^*2*^-propanodeoxyguanosine adducts have also been detected in studies analysing the leukocyte DNA from healthy non-smokers and smokers (Chen 2011; Zhang et al. 2011). Acrolein-DNA adducts were also detected in blood samples of untreated rats and mice as well as in human samples (Nath and Chung 1994; Nath et al. 1996). Zhang et al. (2011) reported that there was no significant difference between the total acrolein-DNA adduct levels in smokers and non-smokers suggesting that glutathione conjugation may effectively remove acrolein from external exposures such as cigarette smoking, protecting leukocyte DNA from damage. These results would imply that levels of these DNA adducts may reflect endogenous rather than exogenous exposure.

Other biomolecular targets for acrolein that have been reported from in vitro studies include amino acid residues, cysteine, histidine, and lysine on proteins. Li et al. reported on a competitive enzyme-linked immunosorbent assay (ELISA) method to detect possible acrolein protein adducts in serum of exposed rats (Li et al. 2004).

#### Tissue biomarkers

Acrolein protein adducts can be assessed using antibody-based techniques, high-performance liquid chromatography (HPLC), and mass spectrometry, and have been demonstrated in vivo in several tissues (Aldini et al. 2011; Moghe et al. 2015).

Acrolein-DNA adducts have also been detected in liver and mammary gland samples of untreated rats, mice and humans (Nath and Chung 1994; Nath et al. 1996).

#### Available PBK studies

Kiwamoto et al. (2015) developed physiologically based kinetic/dynamic models to examine dose-dependent detoxification and DNA adduct formation of a group of 18 food-borne acyclic α,β-unsaturated aldehydes, including acrolein. Acrolein was identified to induce the highest DNA adduct levels among the 18 aldehydes tested, primarily due to its relatively lower detoxification efficiency via oxidation or glutathione *S*-transferase-mediated mediated GSH conjugation. The PBK model predicted level of formation of acrolein GSH conjugates in the liver was only up to 3.3-fold higher than the in vivo reported level excreted as mercapturic acids. At realistic dietary intake, the PBK model predicted formation of 0.036 adducts/10^8^ nt for acrolein in the liver which is orders of magnitude lower than endogenous background levels of structurally similar 1,*N*^*2*^-exocyclic deoxyguanosine adducts observed in disease free human liver.

#### Endogenous formation and accompanying levels of the biomarkers

Acrolein can be formed endogenously as a by-product of certain metabolic pathways such as lipid metabolism, lipid peroxidation, glycolysis, the amino acid turnover or the oxidative deamination of polyamines (Uchida et al. 1998; Stevens and Maier 2008; Guth et al. 2013; Burcham 2017). For example, acrolein may result from water elimination from 2-hydroxypropanal which can be formed via oxidation of the amino acid threonine under oxidative stress or enzymatically by myeloperoxidase. Furthermore, the degradation of polyamines such as spermine and spermidine by copper-dependent amine oxidases can lead to 3-aminopropanal, which in turn can be converted to acrolein by elimination of ammonia (Stevens and Maier 2008; Guth et al. 2013). Acrolein can also be produced during amine oxidase metabolism of endogenous polyamines (Esterbauer et al. 1991; ATSDR 2007; Stevens and Maier 2008; Alwis et al. 2015). Another source for endogenous acrolein production is from myeloperoxidase metabolism of l-threonine by activated human neutrophils (Anderson et al. 1997). Human biomonitoring studies which have detected 3-HPMA in urine of healthy non-smoking human volunteers provide evidence supportive of endogenous production of acrolein (Carmella et al. 2007; Hecht et al. 2015). It is at present not clear to what extent this endogenous formation contributes to the total acrolein exposure, and how to assess exposure from exogenous sources against this endogenous background formation (Guth et al. 2013).

#### Data gaps and research needs

For use of any of the above biomarkers in acrolein exposure assessment definition of correlations between external exposure and levels of the respective biomarkers remain to be established preferably for the human situation. Such correlations will need to take the contribution form endogenous formation of acrolein into account, including factors causing inter-individual differences in both endogenous formation as well as in formation of the various biomarkers at a certain level of exposure. At present it is not clear to what extent this endogenous acrolein formation contributes to the total acrolein exposure, and how to assess the external exposure against this endogenous formation. Urinary 3-HPMA has been used as biomarker for acrolein exposure especially in a qualitative way and its further use to define quantitative acrolein exposure levels is currently hampered by the fact that also other compounds may metabolize to 3-HPMA, which may complicate the definition of quantitative relationships. Levels of DNA adducts in leukocytes may also reflect exposure to acrolein but also for this biomarker the relative contribution from endogenous sources of acrolein needs to be better understood and quantified. This also holds for the various possible tissue biomarkers consisting of amino acid or protein adducts or tissue DNA adducts.

## Discussion and conclusions

From the overview presented above it becomes clear that the field of complementary approaches to exposure assessment of process-related contaminants in food by biomarker monitoring is promising and still under development. The next sections discuss the challenges and future perspectives defined.

### Use of biomarkers in exposure assessment for process-related contaminants

Formation of process-related contaminants including acrylamide, 3-MCPD esters, glycidyl esters, furan and acrolein is well established although some data gaps exist in the identification of the variety of pathways and reactions contributing to their formation in food upon processing. Their intrinsic reactivity and sometimes volatility implies that analytical data generated on products as taken from the shelf do not reflect the amounts ingested by the consumer, and reliable quantitative information on evaporation losses are unavailable and difficult to estimate or generalize.

Deriving accurate consumer exposure estimates for process-related contaminants from food occurrence and consumption data is thus complicated. Use of biomarkers may provide a novel approach to exposure assessment of process-related contaminants in food. At the current state of the art various urinary, blood and/or tissue biomarkers are available that may reflect exposure to process-related contaminants. These biomarkers have already been used to monitor human exposure levels, generally in a qualitative way. Use of biomarker data to inform quantitative exposure assessment may require definition of correlations between quantitative biomarker data and external exposure levels. For use of any of the above biomarkers in exposure assessment definition of correlations between external exposure and levels of the respective biomarkers remain to be established preferably for the human situation.

### Defining correlations between external exposure and biomarker levels

A parameter reflecting a relationship between levels of external exposure and a biomarker levels is the so-called biomonitoring equivalent (BE), defined as the concentration or range of a chemical or its metabolites in a biological medium (blood, urine, or specific tissue) that reflect exposure below existing health-based exposure guidance value such as a Reference Dose (RfD) or Tolerable or Acceptable Daily Intake (TDI or ADI) (Hays and Aylward 2008).

For several compounds, including the process-related contaminants acrylamide and furan, such BE values have been defined (Hays and Aylward 2008, 2009). For acrylamide BEs are calculated especially for the AA haemoglobin valine terminal adduct [*N*-(2 carbamoylethyl)valine (AA-Val)], the GA-haemoglobin valine terminal adduct [*N*-(2-carbamoyl-2-hydroxyethyl)valine (GA-Val)], and the urinary AA mercapturic acid *N*-acetyl-*S*-(2-carbamoylethyl)cysteine (AAMA) (Hays and Aylward 2009).

Another way to translate quantitative biomarker data to quantitative exposure levels may be through the use of PBK modelling. Anatomical features, and physiological and biochemical processes control the ADME of chemicals, including those present naturally in food, or those introduced during manufacturing, processing or cooking. PBK models are one form of biokinetic models historically used by the exposure science, toxicology, risk assessment and drug development communities to relate external exposures to internal exposures or biomonitoring data (e.g. urine levels), or calculate external exposures from internal exposure or biomonitoring data. The use of PBK modelling for exposure assessment has expanded to include reconstruction of exposures. In this particular use, the full available understanding of the biokinetics of a compound, from absorption to a particular exposure biomarker such as a urine or blood concentration is used to convert a biomarker level into an exposure level. For example, for bisphenol A, single spot urine levels can be used to estimate oral exposures by repeatedly running the published PBK model until an exposure level is found that leads to the measured urinary level. This approach has been used for other compounds as well (Louisse et al. 2016). Overall, the value of PBK and other biokinetic modelling approaches for use in exposure assessment should not be overlooked in favour of only experimental methods. PBK models, through their direct representation of kinetic processes and the normal linkages between target sites of exposure and tissues and biofluids that are commonly or increasingly used for biomonitoring (hair, nails, blood, urine, sweat) are the most effective means of integrating exposure data from these disparate sources and linking them to levels of exposure. With respect to the process-related contaminants of the present review the overview presented revealed that for acrylamide, furan and acrolein PBK models relating external dose levels to biomarker levels have been developed. So far, however, these models have not been used for so-called reverse dosimetry, that is, to use the PBK model to predict exposure levels, based on levels of the respective biomarkers. Clearly such approaches enabling the description of quantitative relationships between biomarker levels and exposure levels are essential to further develop the field of biomarker-based exposure assessment and PBK models are expected to play an important role in this methodology, being perhaps more suitable than defining mathematical correlations without including the underlying kinetics. At the present state of the art significant challenges remain for validating the respective biomarkers of dietary exposure to the different process-related contaminants, and PBK models can be of use in this context.

### Duplicate diet studies

Whatever method is used to define the required correlations between external exposure levels and biomarker levels it is of importance to take into account that intrinsic reactivity and volatility resulting in evaporation and other losses upon preparation of the food hamper the exposure assessment and thus also the definition and/or validation of the respective correlations. To solve this issue to the best possible extent and base the correlations to be defined on the quantification of contaminants in the products as “consumed” and not as “produced”, future studies should include so-called duplicate diet studies. A duplicate diet study is a study in which human subjects consume the test diet, but also a duplicate portion of all food as prepared, served and consumed is made available for chemical analysis. Performing duplicate diet studies allow to exactly measure the dietary intake of a given process related contaminant and to compare this with the urinary output of appropriate metabolites that allow biomarker-based dosimetry. EFSA recommended that in order to improve the exposure assessment of acrylamide, duplicate diet studies be conducted and that data on urinary metabolite levels be collected from individuals participating in such studies for the purpose of validating the biomarkers (EFSA 2015). Of the process-related contaminants considered in the present overview such duplicate diet studies were only performed for acrylamide (Ruenz et al. 2016) but were so far not yet used to define quantitative relationships between external dose levels and biomarker levels detected, that would allow predicting external dose levels based on detected biomarker concentrations. A factor complicating the definition of these relationships, even when performing duplicate diet studies appears to be the possible endogenous formation of the process-related contaminants.

### Endogenous formation of process-related contaminants

Observations in animal studies and in human intervention studies under highly controlled conditions have been performed. Minimizing background exposure in these studies revealed that the process-related contaminants under investigation may also be formed endogenously. Clearly this seriously hampers development of methods for exposure assessment based on biomarker quantification, especially in the low dose range where the contribution from endogenous formation may be significant when compared to the contribution from an external source. At the present state-of-the-art experiments are needed to better define the endogenous pathways leading to endogenous formation of the different biomarkers, the actual levels formed as well as influences of inter-individual variation and external factors on this endogenous formation. Adequate definition of these endogenous pathways and the resulting levels of biomarkers may be an important prerequisite for the future risk assessment of process-related contaminants especially when the levels of endogenous formation would be in the range of what is expected to result from levels of normal dietary exposure.

Although levels of DNA adducts are generally considered biomarkers of exposure rather than biomarkers of effect, it is of interest to consider whether dose levels that result in DNA adduct levels within or even below the endogenous background levels would raise a concern. This comparison could even be extended to levels of related DNA adducts to fully appreciate the relevance of the respective biomarker levels in risk assessment. For example the data reviewed above on acrylamide DNA adduct formation revealed that upon single oral AA application in the dose range up to 100 µg/kg bw to rats the resulting tissue DNA adduct levels are considerably lower than reported for human background levels of structurally related N7-dG adducts (Wu et al. 1999; Cheng et al. 2010; Watzek et al. 2012a). Furthermore, a PBK study on acrolein revealed that at realistic dietary intake, the predicted formation of acrolein-DNA adducts in the liver is predicted to be orders of magnitude lower than endogenous background levels of structurally similar 1,*N*^*2*^-exocyclic deoxyguanosine adducts observed in disease free human liver (Kiwamoto et al. 2015).

### Confounding factors

In addition to the endogenous formation of the process-related contaminants, the overview presented also revealed other important confounding factors when considering the use of biomarkers to define external exposure levels. These include the fact that increases in the same biomarker may result from exposure to more than one contaminant. For example DHPMA is a common urinary metabolite of 2-MCPD, 3-MCPD, glycidol and epichlorohydrin (Eckert et al. 2010). This may point at the need for grouping of biomarkers and related exposures especially when the biomarker indicates at a specific biological potential or adverse effect. Also the smoking status of human subjects has been found to affect biomarkers of for example acrolein, acrylamide, furan and glycidyl ester exposure (Landin et al. 1997; Grill et al. 2015). In addition, inter-individual differences in both endogenous formation and in metabolic pathways leading to biomarker formation upon external exposure need to be taken into account when considering extrapolation of biomarker levels to external dose levels. Incorporation of Monte Carlo based modelling approaches to generate distributions of exposure rather than exact exposure values may prove a valid way forward to take these aspects into account. Especially combining Monte Carlo modelling with PBK modelling may prove a way forward to deal with inter-individual differences. The consequences of variability in those parameters that highly influence the conversion of a biomarker level to external dose values by PBK modelling can be evaluated by Monte Carlo modelling.

### Different types of biomarkers

The present review provides also a clear overview of the different types of biomarkers that can be defined and quantified including urinary, blood or tissue derived biomarkers. Future studies need to address the relationships between the different biomarkers to a further extent. Current data indicate that urinary biomarkers like mercapturic acids may be relevant to monitor short term exposure while blood and tissue biomarkers may better reflect longer term exposure. Thus, haemoglobin adducts would be more suitable than urinary mercapturic acids as chronic exposure biomarkers. However, haemoglobin adducts, including for example diHOPrVal, cannot be used as tissue biomarkers for assessing target organ or tissue exposure levels. For cancer risk assessment, tissue biomarkers such as DNA adducts in target tissues are superior to haemoglobin adducts. Including more than one biomarker may facilitate discrimination between endogenous and external levels of exposure and or short and long term exposure. Thus, as an example the combined analysis of DHPMA, being an urinary biomarker for exposure to both 3-MCPD and glycidol, and haemoglobin adducts, which are not considered 3-MCPD biomarkers, may lead to more precise evaluation of glycidyl ester exposure in human covering short and long term exposures.

Clearly also new technologies for biomarker development are emerging and could be used for exposure assessment. There are three areas of development that are particularly relevant to biomonitoring for food process contaminants: novel matrices, non-targeted analytical chemistry (metabolomics), and computational exposure assessment. Conventional biomonitoring has historically focused on easily accessible bio-fluids, principally urine and blood. Recently, interest has grown in the use of other matrices that offer specific benefits. Hair, nails and baby teeth for example, can accumulate chemicals over time, providing an average exposure level for a longer duration. In addition, where these matrices can be sectioned, there is hope that exposure history can be reconstructed. The field of metabolomics, as it pursues global mapping of small organic molecule metabolites, has produced significant advancements in sensitivity, the breadth of chemical space, sample throughput, and supporting methodologies for identifying unknown analytical features. These approaches are equally applicable to food chemicals and their degradation products or metabolites. Of particular relevance to biomonitoring of food contaminants is the ability to survey for hundreds of compounds simultaneously, and possibly identify previously unknown metabolites of important compounds that may serve as better biomarkers of exposure.

Computational exposure science has a long history, but recent advancements and new applications may be important adjunct approaches to traditional biomonitoring. Of particular relevance are approaches that link production/usage levels, physical chemistry, and usage patterns to estimate human external exposure, then apply biokinetic models to estimate biomarker levels in blood or urine. These approaches can be used to estimate biomarker levels, human exposure and help guide decisions on whether or not biomonitoring is feasible. There are opportunities to adapt these approaches to specific use scenarios, for example calculating exposures to chemicals that would result from a particular use (a new can liner) or a new process (heating, etc.).

### Human data

A final issue to consider that is important for future developments in the field of biomarker-based exposure assessments is the availability of human data and models that apply to human exposure. At the current state of the art human data are needed to validate biomarkers, and validate PBK or other models that link levels of biomarkers to external exposure levels in a quantitative way. Including biomarker analysis in future human studies would already greatly advance the data base needed for further development of this approach. Also of great importance is the establishment of a reliable database on human background data, encompassing a broad range of biomarkers linked to exposure to process-related contaminants. Methodology to monitor an ever increasing spectrum of such biomarkers of endogenous formation of the reactive compounds in human body fluids and tissues is already in hand (adductomics). It is crucial to enlarge and enrich this database in order to better put into human health perspective the effects of exposure to a given dietary process related contaminant.

## Conclusions

From the overview presented it becomes clear that the field of complementary approaches to exposure assessment of process-related contaminants in food by biomarker monitoring is still under development. The current state of the art as well as the existing data gaps and challenges for the future were defined. They include (1) definition of correlations between external exposure and levels of the respective biomarkers preferably for the human situation, using PBK modelling and duplicate diet studies; (2) elucidation of the possible endogenous formation of the process-related contaminants and the resulting biomarker levels; (3) the influence of inter-individual variations and how to include that in the biomarker-based exposure predictions; (4) the correction for confounding factors; (5) the value of the different biomarkers in relation to exposure scenarios and risk assessment, and (6) the possibilities of novel methodologies. In spite of these challenges it can be concluded that biomarker-based exposure assessment provides a unique opportunity to more accurately assess consumer exposure to process-related contaminants in food and thus better inform the risk assessment of the exposure to these contaminants.
